# Comparison of *Balanites aegyptiaca* parts: metabolome providing insights into plant health benefits and valorization purposes as analyzed using multiplex GC-MS, LC-MS, NMR-based metabolomics, and molecular networking†

**DOI:** 10.1039/d3ra03141a

**Published:** 2023-07-20

**Authors:** Mohamed A. Farag, Mostafa H. Baky, Ibrahim Morgan, Mohamed R. Khalifa, Robert Rennert, Osama G. Mohamed, Magdy M. El-Sayed, Andrea Porzel, Ludger A. Wessjohann, Nehal S. Ramadan

**Affiliations:** a Pharmacognosy Department, College of Pharmacy, Cairo University Cairo 11562 Egypt mohamed.farag@pharma.cu.edu.eg +011-202-2362245; b Pharmacognosy Department, Faculty of Pharmacy, Egyptian Russian University Badr City Cairo 11829 Egypt; c Department of Bioorganic Chemistry, Leibniz Institute of Plant Biochemistry (IPB) Weinberg 3 Halle (Saale) 06120 Germany; d Global Public Health Institute, American University in Cairo New Cairo Egypt; e Natural Products Discovery Core, Life Sciences Institute, University of Michigan Ann Arbor MI 48109 USA; f Dairy Science Department, National Research Centre Giza 12622 Egypt; g Chemistry of Tanning Materials and Leather Technology Department, National Research Centre Dokki Giza 12622 Egypt

## Abstract

*Balanites aegyptiaca* (L.) Delile (Zygophyllaceae), also known as the desert date, is an edible fruit-producing tree popular for its nutritional and several health benefits. In this study, multi-targeted comparative metabolic profiling and fingerprinting approaches were conducted for the assessment of the nutrient primary and secondary metabolite heterogeneity in different parts, such as leaves, stems, seeds, unripe, and ripe fruits of *B. aegyptiaca* using nuclear magnetic resonance (NMR), ultra-performance liquid chromatography (UPLC-MS), and gas chromatography mass-spectrometry (GC-MS) based metabolomics coupled to multivariate analyses and in relation to its cytotoxic activities. NMR-based metabolomic study identified and quantified 15 major primary and secondary metabolites belonging to alkaloids, saponins, flavonoids, sugars, and amino and fatty acids. Principal component analysis (PCA) of the NMR dataset revealed α-glucose, sucrose, and isorhamnetin as markers for fruit and stem and unsaturated fatty acids for predominated seeds. Orthogonal projections to latent structure discriminant analysis (OPLS-DA) revealed trigonelline as a major distinctive metabolite in the immature fruit and isorhamnetin as a major distinct marker in the mature fruit. UPLC-MS/MS analysis using feature-based molecular networks revealed diverse chemical classes *viz.* steroidal saponins, N-containing metabolites, phenolics, fatty acids, and lipids as the constitutive metabolome in *Balanites*. Gas chromatography-mass spectroscopy (GC-MS) profiling of primary metabolites led to the detection of 135 peaks belonging to sugars, fatty acids/esters, amino acids, nitrogenous, and organic acids. Monosaccharides were detected at much higher levels in ripe fruit and disaccharides in predominate unripe fruits, whereas *B. aegyptiaca* vegetative parts (leaves and stem) were rich in amino acids and fatty acids. The antidiabetic compounds, *viz*, nicotinic acid, and trigonelline, were detected in all parts especially unripe fruit in addition to the sugar alcohol d-pinitol for the first time providing novel evidence for *B. aegyptiaca* use in diabetes. *In vitro* cytotoxic activity revealed the potential efficacy of immature fruit and seeds as cytotoxic agents against human prostate cancer (PC3) and human colorectal cancer (HCT-116) cell lines. Collectively, such detailed profiling of parts provides novel evidence for *B. aegyptiaca* medicinal uses.

## Abstract 2

*Balanites aegyptiaca* (L.) Delile (Zygophyllaceae), also known as the desert date, is an edible fruit-producing tree popular for its nutritional and several health benefits.

## Graphical abstract

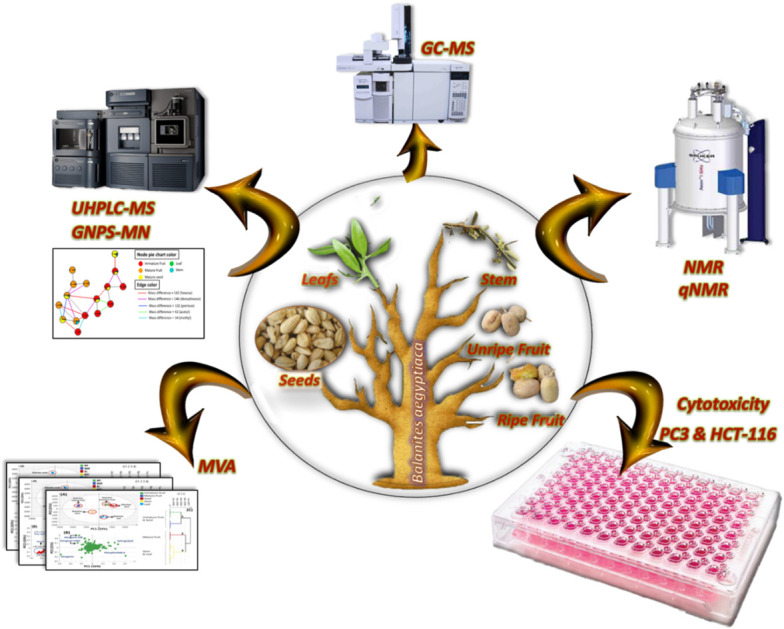

## 1. Introduction

Recently, the growth of consumer demand for herbal supplements and functional foods owing to their medicinal value warranted the development of analytical tools to ensure their quality characteristics and active ingredient identification as novel quality verification techniques.^1^ Metabolite fingerprinting is one of the growing approaches found to be suitable to verify the quality of food-based matrices by profiling their components.^2^*Balanites aegyptiaca* L. belongs to the family Zygophyllaceae, a fruit-producing tree widely distributed in Africa and in particular the tropical and subtropical regions of Africa.^3^*B. aegyptiaca* is also indigenous in Egypt.^4^*B. aegyptiaca* unripe and fully ripe fruits are nutritionally important as sweet and edible and consumed either fresh or processed into commercial beverages.^3^ Moreover, seeds yield fixed oil rich with significant nutrients and could be used for both culinary and biodiesel purposes.^5^

*B. aegyptiaca* is commercially important as its leaves and fruits are used as livestock fodder; timber is used in furniture manufacturer; wood can be used for the production of charcoal for both fuel and industrial purposes; lastly, seed oil is used as biodiesel.^3^ Besides its industrial and food value, *B. aegyptiaca* exerts several health benefits being traditionally used for the treatment of several ailments.^1^*B. aegyptiaca* is reported for its biological importance and benefits human health as being antidiabetic, antioxidant, anti-inflammatory, hepatoprotective, anticancer, and antimicrobial.^3^ Roots and bark are used as emetic, purgative, anthelmintic, and anti-malarial ^1^materials. The fruits are used for jaundice treatment and seed oil as a laxative, antihemorrhoids, stomach aches, jaundice, yellow fever, syphilis, and epilepsy.^3^

With regards to the chemical composition of *B. aegyptiaca*, ripe fruits, leaves, and seeds are rich in carbohydrates, protein, amino acids, vitamins, and fatty acids such as oleic, linoleic, and palmitic.^6^ Several phytochemicals are reported from *B. aegyptiaca* including polyphenols, flavonoids, saponins, coumarins, alkaloids, and steroids.^7^ Such rich chemical makeup in different parts of *Balanites* warrants the need for analytical tools, which can dissect metabolic pathways in each part and prioritize its potential uses based on detailed metabolites profiling.

In recent years, metabolomics is a growing analytical approach increasingly used for the quality assessment of food products to assure their quality in both targeted and untargeted manners.^8^ Metabolomics can be typically employed using either fingerprinting in which extracts are directly analyzed using spectroscopic techniques such as NMR *versus* profiling by performing a chromatographic step pre-detection aiding to improve sensitivity level and metabolites identification score.^9^ Although less sensitive than MS, NMR can provide the standardization of plant extracts using quantitative NMR with no need for standards.^2^ Recently, nuclear magnetic resonance (NMR) as a potent tool has been employed in conjunction with the liquid chromatography linked to MS (LC/MS) approach for the identification of major metabolites in diverse herbal crude extracts as indicated by various 1D and 2D techniques.^10^ Furthermore, ^1^H-NMR is employed for absolute quantitation of major identified metabolites in the specified sample extracts for further standardization objectives where the signal integration of the compound is directly proportional to its molar concentration.^11^ In order to categorize the complexity of obtained NMR datasets, multivariate analyses are frequently employed including unsupervised tools such as principal component analysis (PCA), hierarchical cluster analysis (HCA), and supervised orthogonal projections to latent structures discriminant analysis (OPLS-DA).^9^ The integration of multivariate analysis towards the analysis of spectral datasets provides a more powerful approach for addressing the chemical profile heterogeneity amongst different parts of *Balanites*, besides its quality assessments. For the assessment of metabolites heterogeneity among different parts of *Balanites* and to aid in identifying markers for each, a multiplex approach comparing MS and NMR-based metabolomics was employed for the analysis of stem, seed, leaf and fruits at different maturity stages for the first time. Compared to GC-MS employed for primary metabolites profiling, UHPLC-HRMS/MS was adopted for secondary metabolites profiling considering their polar non-volatile nature, whereas NMR provided standardization of its extracts. This work aimsed to investigate metabolome in the different *B. aegyptiaca* parts *viz.* immature fruit (BIF), mature fruit (BMF), leaf (BL), seed (BS), and stem (BST) in the context of its primary and secondary metabolites accounting for the plant health value. Further, cytotoxicity assessment of *Balanites* was performed in relation to their chemical profiles.

## 2. Materials and methods

### 2.1. Plant materials, chemicals and cell lines

*Balanites aegyptiaca* (L.) Delile samples including immature fruit (BIF), mature fruit (BMF), leaf (BL), seed (BS), and stem (BST) were collected from Wadi El Gemal National Park, Marsa Alam, Red Sea Governorate in Egypt in January 2022, and the plant was botanically identified by Eng. Ahmed Abdel-Razek, Senior Environmental Researcher Wadi El-Gamal-Hamata National Park (WGNP).

Hexamethyldisiloxane (HMDS) and methanol-d_4_ (99.80% D) were supplied by Deutero GmbH (Kastellaun, Germany). Formic acid and acetonitrile of LC-MS grade were obtained from J. T. Baker (The Netherlands), and Milli-Q water was used for LC analysis. All other standards as well as chemicals were obtained from Sigma Aldrich (St. Louis, MO, USA). Two human cell lines were used to determine the anti-proliferative and cytotoxic activity of the extracts, namely PC-3 prostate cancer and HCT-116 colorectal cancer cells. Both cell lines were obtained from DSMZ (Braunschweig, Germany) by the Leibniz Institute of Plant Biochemistry (IPB), where all cell-based assays were conducted. Cell culture materials including the basal media RPMI 1640 and McCoy's 5A, phosphate-buffered saline (PBS), 0.05% trypsin–EDTA, and fetal calf serum (FCS) were purchased from Capricorn Scientific (Ebsdorfergrund, Germany). For the cell viability and cytotoxicity assays, 3-(4,5-dimethylthiazol-2-yl)-2,5-diphenyltetrazolium bromide (MTT), crystal violet (CV) and paraformaldehyde (PFA) were used from Sigma Aldrich (St. Louis, MO, USA). The acetic acid solution was purchased from Carl Roth (Karlsruhe, Germany).

### 2.2. Sample preparation for NMR and MS analyses

The sample preparation was following the protocol described in Zayed *et al.*^12^ In brief, 120 mg of each dried organ powder was homogenized with 5 mL 100% methanol with 10 μg mL^−1^ of umbelliferone as an internal standard using a Turrax mixer (9469×*g*) for five 20 s periods, with 1 min interval to avoid warming. Afterwards, vigorous centrifugation (3000×*g* for 30 min) was performed to remove plant debris.

#### 2.2.1. NMR analysis

4 milliliters aliquots were obtained with a syringe for analysis, and the solvent was evaporated under nitrogen until dry. Upon drying, the extracts were then suspended with 800 μL 100% methanol-d_4_ containing 0.94 mM hexamethyldisiloxane (HMDS) as an internal NMR standard. The centrifuged supernatant at 3000×*g* for 1 min was transferred to a 5 mm NMR tube. For NMR quantification and calibration of chemical shift, HMDS was added to a final concentration of 0.94 mM. All ^1^H-NMR spectra for MVA were collected within 48 hours, with instantly prepared samples prior to data acquisition. Control trials repeated after 48 hours revealed no significant variance.

#### 2.2.2. High-resolution UPLC-MS analysis

0.5 milliliters prepared as previously described in Section (2.2 Sample preparation for NMR and MS analyses) were aliquoted for UPLC-MS post-filtration using a 0.22 μm filter. Analysis conditions reported by Younis *et al.* were followed.^13^ Characterization of the individual peak was established by the generation of each candidate formula with its mass accuracy limit of <10 ppm considering mainly RT, MS^2^ data, and the reported reference literature and Phytochemical Dictionary of Natural Products Database, CRC, Wiley. The HPLC-MS analysis was performed using an Agilent single quad and a Poroshell 120 EC-C18 column of dimensions 2.7 μm 4.6 × 50 mm, a flow rate of 0.8 mL min^−1^, mobile phases of 0.1% formic acid in water (solvent A) and 0.1% formic acid in acetonitrile (solvent B), and a gradient as follows: 1 min, 0.5% B; 1–20 min, 0.5–100% B; 20–25 min, 100% B. High-resolution tandem mass spectrometry coupled with high-performance liquid chromatography (HR-LC-MS/MS) was performed using an Agilent QTOF and a Poroshell 120 EC-C18 column of dimensions 2.7 μm 2.1 × 100 mm, a flow rate of 0.25 mL min^−1^, mobile phases of 0.1% formic acid in water (solvent A), 0.1% formic acid in acetonitrile (solvent B), and a gradient as follows: 1 min, 0.5% B; 1–20 min, 0.5–100% B; 25–30 min, 100% B. Identification was based on a comparison of MS spectra, and MS/MS spectral data with that reported in the literature and the Dictionary of Natural Products database (CRC, Wiley).^14,15^

#### 2.2.3. GC-MS analysis

100 μL of the methanol extract was taken in screw-cap vials and left to evaporate under a nitrogen gas stream until complete dryness. For derivatization, 150 μL of *N*-methyl-*N*-(trimethylsilyl)-trifluoroacetamide (MSTFA) previously diluted 1/1% with anhydrous pyridine was mixed with the dried methanol extract and incubated for 45 min at 60 °C prior to analysis using GC-MS. Separation of silylated derivatives was achieved on an Rtx-5MS (30 m length, 0.25 mm inner diameter, and 0.25 m film).^8^ Three biological replicates were extracted and examined in parallel for each specimen under the same conditions. For biological variance assessment, within each specimen and analysis condition, three independent biological replicates were simultaneously analyzed under the same conditions. GC-MS analysis was performed on an Agilent 5977B GC/MSD equipped with a DB-5 column (30 m × 0.25 mm i.d. × 0.25 μm film thickness; Supelco) and coupled to a quadrupole mass spectrometer. The interface and the injector temperatures were both set at 220 °C. Volatile elution was carried out using the following gradient temperature program: oven was set at 40 °C for 3 min, then increased to 180 °C at a rate of 12 °C min^−1^, kept at 180 °C for 5 min, finally increased at a rate of 40 °C min^−1^ to 240 °C and kept at this temperature for 5 min. Helium was utilized as a carrier gas with a total flow rate of 0.9 mL min^−1^.

### 2.3. NMR data acquisition

All spectra were recorded on an Agilent VNMRS 600 NMR spectrometer operating at a proton NMR frequency of 599.83 MHz using a 5 mm inverse detection cryoprobe, digital resolution 0.367 Hz per point (32k complex data points), pulse width (pw) = 2.1 μs (30°), relaxation delay = 18 s, acquisition time = 2.0 s, number of transients = 160, and temperature = 297 K. Zero filling up to 128 K and an exponential window function with lb = 0.4 was used prior to Fourier transformation. The 2D-NMR spectra were recorded at a frequency of 599.83 MHz using standard CHEMPACK 6.2 pulse sequences (TOCSY and HSQC) implemented in standard VNMRJ 4.0 A spectrometer software. The HSQC experiment was optimized for ^1^*J*_CH_ = 146 Hz with DEPT-like editing and ^13^C-decoupling during the acquisition time.

### 2.4. NMR data processing and multivariate data analyses

Spectra were imported to Mnova Mestrelab Research version 14 software. The spectra were referenced to internal HMDS at 0.062 ppm for ^1^H-NMR. Spectral intensities were reduced to integrated regions, referred to as buckets, of equal width (0.04 ppm) for all spectral (*δ* 0.4–10.0 ppm) and aromatic (*δ* 5.5–10.0 ppm) regions. The spectral regions corresponding to the residual solvent signals, *δ* 5.45–5.87 ppm (water), *δ* 3.33–3.28 ppm (methanol) and *δ* 7.62–7.42, *δ* 8.85–8.69 ppm (impurities), were removed before multivariate analyses. This binning allowed the evaluation of the absolute quantification of the identified metabolites.

SIMCA-P version 13.0 (Umetrics, Umea, Sweden) was used to import the bucket table. Hierarchical cluster analysis (HCA), principal component analysis (PCA), and orthogonal projection to latent structure-discriminant analysis (OPLS-DA) were performed with all variables mean-centered and scaled to Pareto variance. The seven-fold cross-validation approach was used to determine the optimal number of main components requisite for data modelling, and the distance to the model (DModX) test was employed to confirm the presence of outliers. Permutation tests, receiver operating characteristic (ROC) curves, and CV-ANOVA were used to verify the constructed OPLS-DA models (ANOVA of cross-validated residuals). The *S*-plot, which was reported with covariance (*p*) and correlation (*p*_cor_), besides, the variable influence in the projection (VIP) was then analyzed to identify metabolite markers (VIP).

### 2.5. Quantification of major metabolites *via*^1^H-NMR

The peak area of the selected proton signals pertaining to the target compounds and the peak area of the internal standard (HMDS) were manually integrated for all samples for the quantification of metabolites indicated in Tables 1 and 2 using the NMR technique.

**Table 1:** Resonance assignments with chemical shifts of constituents identified in 600 MHz ^1^H-NMR, HSQC and TOCSY of different parts of *Balanites aegyptiaca* methanol extracts

Metabolite	Assignment	*δ* ^1^H (ppm)	*δ* ^13^C in HSQC (ppm)	TOCSY correlations *δ*^1^H (ppm)	Parts
**Alkaloids**					
Trigonelline (N1)	C-2	9.3 (s)	149.2	—	BIF, BMF, BL, BS, BST
	C-4	8.95 (d, *J* = 8.0 Hz)	145.6	9.02 (H-6), 8.14 (H-5)	
	C-5	8.14 (dd, *J* = 8.0, 6.0 Hz)	128.6	8.95 (H-4), 9.02 (H-6)	
	C-6	9.02 (d, *J* = 6.1 Hz)	144.9	8.14 (H-5)	
	N–CH_3_	4.4 (s)	nd	—	

**Saponins**					
Diosgenin (N2)	C-3	5.02 (m)	82.9	4.58 (H-6)	BIF, BMF, BL, BS, BST
	C-6	4.58 (dd, *J* = 5.5, 1.98 Hz)	121.2	5.02 (H-3)	
	C-18	0.8 (s)	18.03	—	
	C-19	1.02 (s)	21.2	—	
	C-27	0.78 (d, *J* = 6.3 Hz)	17.5	—	

**Flavonoids**					
Isorhamnetin (N3)	C-6	6.25 (d, *J* = 1.92 Hz)	101.5	6.51 (H-8)	BMF, BL, BS, BST
	C-8	6.51 (d, *J* = 1.92 Hz)	nd	6.25 (H-6)	
	C-3′ (OCH_3_)	3.70^a^ (s)	56.5	Overlapped	
	C-5′	6.74 (d, *J* = 8.52 Hz)	nd	7.13 (H-6′)	
	C-6′	7.13 (br.d, *J* = 8.52 Hz)	121.6	6.74 (H-5′)	

**Sugars**					
α-Glucose (N4)	C-1	5.19 (d, *J* = 3.72 Hz)	95.2	3.46 (H-2), 3.70 (H-3)	BIF, BMF, BL, BS, BST
	C-2	3.46^a^ (m)	75	3.70 (H-3)	
	C-3	3.70^a^	76.2	Overlapped	
β-Glucose (N5)	C-1	4.41 (d, *J* = 5.88 Hz)	100.2	nd	BIF, BMF, BL, BS, BST
	C-6	3.64	65.5	Overlapped	
Galactose (N6)	C-1	4.31 (d, *J* = 7.34 Hz)	105.9	3.46 (H-2)	BLF, BLMF, BL, BS, BST
	C-2	3.46^a^	75	4.31 (H-1)	
Sucrose (N7)	C-1	4.66 (br s)	91.6	3.50 (H-2), 3.70 (H-3)	BIF, BMF, BL, BS, BST
	C-2	3.50 (dd, *J* = 3, 9.9 Hz)	79.2	Overlapped	
	C-3	3.70	74.7	Overlapped	
	C-6	3.87 (d, *J* = 1.93 Hz)	63.7	nd	
	C-2′	4.13^a^ (br s)	79.1	3.70 (H-4′ and H-5′)	
	C-3′	4.13^a^	78.0	3.70 (H-4′ and H-5′)	
	C-4′	3.70^a^ (s)	83.7	Overlapped	
	C-5′	3.70^a^ (s)	62.0	Overlapped	
	C-6′	3.65 s	65.4	nd	

**Amino acids/nitrogenous compounds**					
Alanine (N8)	C-2	3.59^a^	52.1	1.54 (H-3)	BIF, BIMF, BL, BS, BST
	C-3	1.54 (d, *J* = 7.3 Hz)	18.4	3.59 (H-2)	
Valine (N9)	C-4	1.04 (d, *J* = 6.96 Hz)	19.4	Overlapped	BIF, BIMF, BL, BS, BST
	C-5	1.08 (d, *J* = 6.96 Hz)	20.1	Overlapped	
Phenylalanine (N10)	C-2	3.80 (m)	58.5	Overlapped	BIF, BIMF, BL, BS, BST
	C-4′	7.34^a^	128.3	7.30 (H-3′ and H-5′), 7.27 (H-2′ and H-6′)	
	C-3′/C-5′	7.30^a^	128.3	Overlapped	
	C-2′/C-6′	7.27^a^	128.3	Overlapped	
Threonine (N11)	C-2	3.74^a^	61.6	1.24 (H-4), 4.16 (H-3)	BIF, BMF, BL, BS, BST
	C-3	4.16^a^	67.7	3.74 (H-2), 1.24 (H-4)	
	C-4	1.24 (d, *J* = 6.33 Hz)	19.3	3.74 (H-2), 4.16 (H-3)	
Choline (N12)	N–(CH_3_)_3_	3.25 (s)	55.9	—	BLF, BLMF, BL, BS, BST
	N–CH_2_	3.40	72.9	4.22 (O–CH_2_)	
	O–CH_2_	4.22 m	63.3	3.40 (N–CH_2_)	

**Fatty acids**					
ω-9, ω-6, ω-3 fatty acids	Olefinic carbons	5.30–5.35 (m)	nd	2.81 (bis-allylic CH_2_), 2.01 (allylic CH_2_)	BIF, BMF, BL, BS, BST
	Allylic CH_2_	2.01 (m)	nd	1.28 (CH_2_)*n*, 5.30 (olefinic CH)	
ω-9 oleic acid (N13)	*t*-CH_3_	0.89 (t, 6.96 Hz)	15.65	1.28 (CH_2_)*n*	BLF, BLMF, BL, BS, BST
	(CH_2_)*n*	1.28 (br s)	31.7	0.89 (*t*-CH_3_), 1.60 (H-3), 2.29 (H-2), 2.01 (allylic CH_2_)	
	C-2	2.29 (t, 7.44 Hz)	nd	1.28 (CH_2_)*n*, 1.60 (H-3)	
	C-3	1.60 (m)	27.3	1.28 (CH_2_)*n*, 2.29 (H-2)	
ω-6 fatty acid (N14)	Bis-allylic CH_2_	2.77 (br s)	nd	5.33 (olefinic CH)	BIF, BMF, BL, BS, BST
ω-3 fatty acid (N15)	*t*-CH_3_	0.94 (t, *J* = 7.4 Hz)	15.65	nd	BIF, BMF, BL, BS, BST
	Bis-allylic CH_2_	2.81 (t, *J* = 4.5 Hz)	27.7	5.33 (olefinic CH)	

a Overlapped.

b nd, not detected; BIF, *Balanites* immature fruit; BMF, *Balanites* mature fruit; BL, *Balanites* leaf; BS, *Balanites* seed; BST, *Balanites* stem.

**Table 2:** ^1^H-NMR quantification of most primary and secondary metabolites detected in different samples of *Balanites*. Values are expressed as μg per mg dry powder ± S.D (*n* = 3), see Experimental section. Chemical shifts used for metabolite quantification were determined in methanol-*d*_6_ and expressed as relative values to HMDS (0.94 mM final concentration)

Metabolite	Protons used in quantification	BIF	BMF	BL	BS	BST
Trigonelline	H-2	1.08 ± 0.05	0.2 ± 0.01	0.7 ± 0.1	0.3 ± 0.06	0.4 ± 0.2
Diosgenin	H-3	Overlap	Overlap	2.5 ± 1.5	4.7 ± 0.9	0.4 ± 0.2
Isorhamnetin	H-6	nd^a^	0.2 ± 0.03	1.3 ± 0.7	0.5 ± 0.2	0.09 ± 0.05
α-Glucose	H-1	0.02 ± 0.002	0.2 ± 0.008	0.003 ± 0.004	0.02 ± 0.0007	0.01 ± 0.003
β-Glucose	H-1	0.08 ± 0.002	0.03 ± 0.005	0.007 ± 0.001	0.04 ± 0.01	0.005 ± 0.003
Galactose	H-1	0.02 ± 0.001	Overlap	0.003 ± 0.0004	0.02 ± 0.0007	0.001 ± 0.0006
Sucrose	H-1	0.8 ± 0.4	2.9 ± 0.9	0.6 ± 0.3	0.2 ± 0.4	0.3 ± 0.4
Alanine	H-3	Overlap	Overlap	2.08 ± 0.5	2.8 ± 0.3	0.8 ± 0.07
Valine	H-5	6.1 ± 0.3	Overlap	2.08 ± 0.2	2.7 ± 0.1	0.7 ± 0.04
Phenylalanine	H-3′, H-4′, H-5′	Overlap	Overlap	0.4 ± 0.04	Overlap	0.2 ± 0.1
Threonine	H-4	10.6 ± 0.9	14.2 ± 0.4	3.9 ± 0.5	6.2 ± 0.4	1.2 ± 0.2
Choline	N–(CH_3_)3	0.4 ± 0.03	1.3 ± 0.1	0.3 ± 0.05	0.7 ± 0.05	0.2 ± 0.01
ω-9 fatty acid	*t*-CH_3_	12.7 ± 0.08	25.6 ± 2.1	17.9 ± 1.3	89.7 ± 1.6	10.7 ± 0.6
ω-3 fatty acid	Bis-allylic CH_2_	Overlap	9.3 ± 0.3	3.2 ± 1.1	Overlap	1.5 ± 0.4
ω-6 fatty acid	Bis-allylic CH_2_	9.5 ± 1.8	13.7 ± 0.4	Overlap	44.7 ± 1.6	0.3 ± 0.2

a nd: not detected.

The following eqn (1) was applied for the calculations and the protocol was adopted from:^12^1
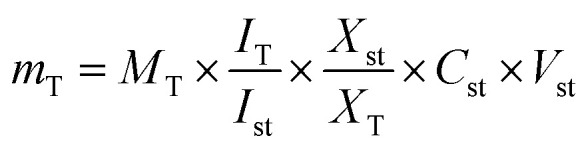


*m*_T_: mass of the target compound in the solution used for ^1^H-NMR measurement in μg. *M*_T_: molecular weight of the target compound (g mol^−1^). *I*_T_: relative integral value of the ^1^H-NMR signal of the target compound. *I*_St_: relative integral value of the ^1^H-NMR signal of the standard compound. *X*_St_: number of protons belonging to the ^1^H-NMR signal of the standard compound. *X*_T_: number of protons belonging to the ^1^H-NMR signal of the target compound. *C*_St_: concentration of the standard compound in the solution used for ^1^H-NMR measurement (mmol L^−1^). *V*_St_: volume of the solution used for ^1^H-NMR measurement in mL.

### 2.6. Analysis, modelling and quantification of UPLC/PDA/MS dataset

#### 2.6.1. Data pre-processing using MZmine 2

The acquired MS/MS data were converted from Agilent MassHunter data files (.d) to mzXML file format using MSConvert software,^16^ part of the ProteoWizard package. The. mzXML files were imported and processed with MZmine 2 v2.53,^17^ with the following workflow: (i) mass detection: MS^1^ noise level, 1 × 10^4^; MS^2^ noise level, 1 × 10^2^. (ii) ADAP chromatogram builder: MS-level, 1; min group size in no. of scans, 2; group intensity threshold, 2 × 10^4^; min highest intensity, 5 × 10^3^; *m*/*z* tolerance, 0.05 *m*/*z*. (iii) Chromatogram deconvolution: noise amplitude algorithm: minimum peak height, 2 × 10^4^; the amplitude of noise, 5 × 10^3^ (iv) isotopic peaks grouper: *m*/*z* tolerance, 0.01 *m*/*z*; RT tolerance, 0.05 min; monotonic shape, yes; maximum charge, 2; representative isotope, lowest *m*/*z*. (v) Peak alignment: *m*/*z* tolerance, 0.02 *m*/*z*; weight for *m*/*z*, 75; RT tolerance, 0.2 min; weight for RT, 25. (vi) Peak list rows filter: only features with accompanying MS^2^ data and their retention time between 0 and 15.0 min were kept. (vii) Duplicate peak filter: filter mode, old average; *m*/*z* tolerance, 0.02 *m*/*z*; RT tolerance, 0.25 min. (viii) Fragment search module: RT tolerance, 0.4 min; *m*/*z* tolerance for MS^2^ data, 0.01 *m*/*z*; max fragment peak height, 300%; min MS^2^ peak height, 1 × 10^3^. (ix) Adduct search module: RT tolerance, 0.2 min; search for [M + Na]^+^ adduct; *m*/*z* tolerance, 0.001 *m*/*z*; peak height, 100%. The resulting feature lists were exported to the global natural products social molecular networking (GNPS)-compatible format, using the dedicated “export for GNPS” built-in options.

#### 2.6.2. GNPS feature-based molecular MS/MS network

Using the feature-based molecular networking (FBMN) workflow (version release_28.2)^18^ on GNPS, a molecular network was created. The resulting aligned list of features was exported in an mgf file besides their feature quantification table in CSV format. The values of the feature quantification table were uploaded onto the FBMN page of GNPS. MS^2^ spectra were filtered, all MS/MS fragment ions within ±17 Da of the precursor *m*/*z* were removed, and only the top 6 fragment ions in the ±50 Da window through the spectrum were utilized. The precursor and fragment ion masses were both set to 0.02 Da. Edges of the molecular network were filtered to have a cosine score above 0.7 and more than 5 matched peaks between the connected nodes. The edges between two nodes were kept in the network only if each of the nodes appeared in the other's respective top 10 most similar nodes. The size of clusters in the network was set to a maximum of 100. The molecular networks were visualized using Cytoscape 3.9.1.^17^ The molecular networking job with the edge annotation table is accessible by the following link (https://gnps.ucsd.edu/ProteoSAFe/status.jsp?task=65bb3c3d9d4b412aa243280075e34e1c). Metabolites were annotated based on the molecular formula and their fragmentation pattern, compared to earlier reported data aided with public literature, libraries, and databases. Therefore, further molecular networking, developed using the GNPS system was imported to Cytoscape 3.9.1 to visualize MS/MS data.

#### 2.6.3. Multivariate data analyses (PCA and OPLS)

Relative quantification of metabolic profiles of *B. aegyptiaca* different parts was performed post UPLC-MS, using XCMS data analysis software, freely downloaded as an R package from the Metlin Metabolite Database (Otify, El-Sayed, Michel, and Farag, 2019). PCA and OPLS-DA were performed using the SIMCA-P version 13.0 software (Umetrics, Umeå, Sweden) with all variables mean-centered and scaled to Pareto variance.

### 2.7. GC-MS profiling and modelling of silylated primary metabolites

The protocol to validate silylation was adopted as previously reported.^19^ Soluble sugars, amino acids, organic acids, and fatty acids were quantified using standard curves of glucose, glycine, citric and stearic acids and results were expressed as mg g^−1^. Four serial dilutions were prepared from 10 to 600 μg mL^−1^ for establishing the standard curves. Calibration curves for glucose, glycine, citric acid, and stearic acids displayed a 0.9948 correlation coefficient.^20^

Identification of components was performed by comparing their retention indices (RI) in relation to *n*-alkanes (C6–C20), mass matching to NIST, WILEY library database, and standards if available. Peaks were first deconvoluted using AMDIS software (https://www.amdis.net/) accessed on 28 November 2019 (ref. 8) before mass spectral matching. Data were then subjected to principal component analysis (PCA), hierarchical clustering analysis (HCA), and partial least squares discriminant analysis (OPLS-DA) using SIMCA-P version 13.0 software package (Umetrics, Umeå, Sweden). Markers were subsequently identified by analyzing the *S*-plot, which was declared with covariance (*p*) and correlation (*p*_cor_). All variables were mean-centered and scaled to Pareto variance. Model validation was assessed by computing the diagnostic indices, *viz. Q*^2^ and *R*^2^ values, and permutation testing.

### 2.8. Cytotoxicity assay

#### 2.8.1. Cell culture

PC-3 cells were cultured under standard growth conditions (37 °C and 5% CO_2_ in a humidified atmosphere) in RPMI 1640 medium supplemented with 10% (v/v) heat-inactivated FCS and 2 mM l-glutamine, while for HCT-116 McCoy's 5A medium supplemented with 10% (v/v) heat-inactivated FCS and 2 mM l-glutamine was used.^21^ Cells were monitored daily using light microscopy and were passed whenever the confluency reached 90%. For passaging, the adherent cells were detached with 0.05% trypsin–EDTA for 3 min at 37 °C. After detachment, a 9-fold volume of FCS-containing complete medium was added to stop the trypsin enzyme activity, and the cell suspension was collected and centrifuged for 3 min at 800 rpm. The supernatant was discarded, and the cells pellet was washed once with PBS. Afterwards, the cells were either diluted 10-folds and transferred to a new T75 culture flask in 10 mL of the respective complete culture medium, or cell seeding for cell viability assays was performed in 96-well plates. For seeding, cells were counted using a countess II cell counter (Invitrogen, Waltham, MA, USA). For PC-3, a cell suspension was prepared with a density of 6 × 10^3^ cells per 100 μL, while for HCT-116, 5 × 10^3^ cells per 100 μL were used. Finally, 100 μL per well of these cell suspensions were pipetted in 96-well plates and allowed to adhere overnight under standard growth conditions.

#### 2.8.2. Cell viability assays

Anti-proliferative and cytotoxic effects of the plant extracts of interest were investigated by performing spectroscopic MTT and CV assays, respectively. For that purpose, the cancer cells – seeded as described above – were treated with final concentrations of 200, 100, 50, 25, 12.5, 6.25, 3.125, and 1.5625 μg mL^−1^ of the extracts for 48 h under standard growth conditions. Negative and positive controls were included at each assay plate, *i.e.*, extract-free complete medium was used as a negative control, while digitonin (a highly cytotoxic steroidal saponin) in a final concentration of 150 μM was used as a positive control.^22^

After 48 h incubation, in the case of MTT assay, the treatment solution was discarded, and the cells were washed with 50 μL per well of PBS. Afterwards, the cells were stained with 50 μL of MTT working solution (0.5 mg MTT per mL in RPMI 1640) for 30 min. Subsequently, the staining solution was discarded, and the formazan dye – converted by the viable cell fraction – was dissolved using DMSO. Finally, the formazan absorbance was measured at 570 nm (and background at 670 nm for data normalization) by using a SpectraMax iD5 multi-well plate reader (Molecular Devices, San Jose, CA, USA).^23^ For CV assay, cells were washed with 50 μL per well of PBS, followed by cell fixation using 4% (w/v) PFA in PBS for 10 min. After fixation, the PFA solution was discarded, and the plates were dried for another 10 min. For CV staining, cells were incubated with 50 μL per well of CV solution (0.1% (w/v) in PBS) for 15 min. Afterwards, the cells were washed with ddH_2_O and dried overnight. Subsequently, the CV stain was dissolved using 33% (v/v) acetic acid in ddH_2_O, and absorbance was measured with the SpectraMax iD5 using the aforementioned parameters as well.^24^ Data were determined in three independent biological replicates, each with technical quadruplicates. IC_50_ curves and values of the extracts were calculated by using GraphPad Prism® version 8.0.2 using sigmoidal dose–response curve fitting (variable slope, four parameters).

### 2.9. Statistical analysis

Duplicate samples of each *Balanites* part for each treatment/period were taken. Each sample was analyzed individually in triplicate. The data are presented as mean (standard deviation of three determinations. A probability value of *P* < 0.05 was considered to denote the statistically significant differences.

## 3. Results and discussion

To provide a comparative assessment of datasets derived from various metabolomic platforms including NMR, UPLC-MS, and GC-MS metabolomics, a one-pot extraction method was developed (see Section 2.2 Sample preparation for NMR and MS analyses) was established. Chemometric tools were employed to categorize samples, assure analytical robustness, establish similarities as well as differences amongst samples, and lastly compare the different analytical platforms based on the model's power for the classification potential for the first time in *Balanites*.

### 3.1. NMR fingerprinting of *Balanites* parts

NMR-based metabolomics fingerprinting is a well-established technique for the identification and quantification of metabolites in crude extracts providing a readout of metabolite classes abundance in extracts owing to its universal detection.^25^ A total of 15 metabolites were identified based on 1D- and 2D-NMR analyses (Fig. S1†) and listed alongside their chemical shifts as well as their distribution amongst the different *Balanites* parts in Table 1. The peak assignments were identified according to the previously reported literature,^4^ besides the further confirmation of signal assignments using 2D-NMR experiments including ^1^H–^13^C HSQC (heteronuclear single quantum coherence spectroscopy) and ^1^H–^1^H TOCSY (total correlation spectroscopy). Two regions mainly appeared for all parts in the ^1^H-NMR spectra (Fig. S1†): an up-field region (*δ* 0.5 to 5.5 ppm) mostly relevant to primary metabolites *viz.* saponins (N2), sugars (N4–N7), amino acids (N8–N11), nitrogenous compounds (N12) and fatty acids (N13–N15) and a down-field region (*δ* 5.5–9.5 ppm) assigned to secondary metabolites, *i.e.*, alkaloids (N1), flavonoids (N3), and phenylalanine (N10).

#### 3.1.1. Alkaloids

Four characteristic aromatic signals were assigned at *δ* 9.3 (1H, s), *δ* 8.95 (1H, d, *J* = 8.0 Hz), *δ* 8.14 (1H, dd, *J* = 8.0, 6.0 Hz) and *δ* 9.02 (1H, d, *J* = 6.1 Hz) besides a methyl proton singlet at *δ* 4.4 corresponding to a methyl group at N-1, revealed the existence of the pyridine containing alkaloid; trigonelline (N1).^1^ These signals corresponded to H-2, H-4, H-5, and H-6 protons, respectively, further supported *via*^1^H–^1^H TOCSY and showing HSQC cross-peak correlation to their respective carbons at *δ* 149.2 (C-2), 145.6 (C-4), 128.6 (C-5) and 144.9 (C-6), consistent with previously reported data in the literature^26^ (Table 1). Trigonelline is amongst the major alkaloids isolated from *B. aegyptiaca* contributing to the slight bitterness in *Balanites* fruit and reported to possess antidiabetic and anticancer activities.^27,28^

#### 3.1.2. Saponins

Diosgenin as a major steroidal sapogenin (N2) was detected in all *Balanites* parts, which could be readily distinguished from chemical shifts at *δ* 4.58 ppm (dd, *J* = 5.5, 1.98 Hz; H-6), *δ* 5.02 ppm (m; H-3) confirmed with ^1^H–^1^H TOCSY map as well as HSQC cross-peaks revealing signals at *δ* 121.2 and 82.9 ppm corresponding to C-6 and C-3, respectively. Further, three methyl groups were detected at *δ* 0.8 ppm (s, C-18 methyl), *δ* 1.02 ppm (s, C-19 methyl) and *δ* 0.78 ppm (d, *J* = 6.3 Hz C-27 methyl) with their respective carbons detected at *δ* 18.03 ppm (C-18), *δ* 21.2 ppm (C-19) and 17.5 ppm (C-27) revealed from key HSQC correlations. These data were consistent with previously reported data of diosgenin^29,30^ (Table 1 and Fig. S1†). Diosgenin is reported for its anticancer and antidiabetic activities besides being a substrate for the synthesis of several steroidal drugs.^31,32^

#### 3.1.3. Flavonoids

Isorhamnetin reported for its several health effects alongside its glycosides is amongst the most prominently occurring flavonoids in *Balanites*.^3,33^ Isorhamnetin (N3) was identified from peaks at *δ* 6.25 ppm (d, *J* = 1.9 Hz; H-6) with its respective carbon detected at *δ* 101.5 ppm (C-6), *δ* 6.51 ppm (d, *J* = 1.9 Hz; H-8), *δ* 6.74 ppm (d, *J* = 8.5 Hz; H-5′) and *δ* 7.13 ppm (br.d, *J* = 8.5 Hz; H-6′) with its respective carbon detected at *δ* 121.6 ppm (C-6′). Furthermore, an upfield chemical shift was detected at *δ* 3.70 ppm (3H, s, OCH_3_) with its respective carbon detected at *δ* 56.5 ppm *via* HSQC correlation (Table 1 and Fig. S1†) confirming isorhamnetin structure and in agreement with previously reported data.^34^

#### 3.1.4. Sugars

The chemical shifts detected at *δ* 5.19 ppm (d, *J* = 3.7 Hz), 4.41 ppm (d, *J* = 5.9 Hz), 4.31 ppm (d, *J* = 7.3 Hz), and 4.66 ppm (br s) were readily assigned to anomeric protons of α-glucose (N4), β-glucose (N5), galactose (N6) and sucrose (N7), respectively, and verified from HSQC cross-peak correlations detected at *δ* 95.2, 100.2, 105.9 and 91.6 ppm, respectively.

#### 3.1.5. Amino acids/nitrogenous compounds

Amino acids and nitrogenous compounds represented by alanine (N8), valine (N9), threonine (N11), choline (N12) predominated in all parts. In detail, alanine (N8), valine (N9), threonine (N10) and choline (N12) exhibited clear methyl group signals at *δ* 1.54 ppm (d, *J* = 7.3 Hz, H-3), *δ* 1.04 and 1.08 (d, *J* = 6.9 Hz, H-4 and 5), *δ* 1.24 (d, *J* = 6.3 Hz, H-4) and *δ* 3.25 (s, N–(CH_3_)_3_) ppm, respectively, and showing further respective HSQC cross-peak correlations at *δ* 18.4 (C-3) ppm for alanine (N8), *δ* 19.4 (C-4) and 20.1 (C-5) ppm for valine (N9), *δ* 19.3 (C-4) ppm for threonine (N10) and *δ* 55.9 (N–(CH_3_)_3_) ppm for choline (N12) (Table 1 and Fig. S1†). Detection of phenylalanine (N10) was detected *via* three unique proton chemical shifts of the phenyl ring at *δ* 7.27 (H-2′/H-6′), 7.30 (C-3′/C-5′), and 7.34 (H-4′) ppm with respective HSQC cross-peak correlations at *δ* 128.3 ppm (Table 1 and Fig. S1†). All previous assignments were further confirmed *via* the ^1^H–^1^H TOCSY map (Fig. S3†).

#### 3.1.6. Fatty acids

The signals of olefinic protons resonating at *δ* 5.30–5.35 (m) ppm are considered a key feature for the presence of unsaturated fatty acids together with the allylic methylene at *δ* 2.01 ppm consistent with the ^1^H–^1^H TOCSY correlations (Table 1). The presence of ω-9 fatty acid (N13) and ω-3 fatty acid (N15) was evident from chemical shift at *δ* 0.89 ppm (t, *J* = 6.9 Hz, *t*-CH_3_), *δ* 0.94 ppm (t, *J* = 7.4 Hz, *t*-CH_3_), respectively, with their respective carbons detected at *δ* 15.65 ppm. Bis-allylic methylene of ω-6 (N14) and ω-3 (N15) fatty acids were detected at *δ* 2.77 ppm (br s) and 2.81 (t, *J* = 4.5 Hz) ppm, respectively. All assignments were further confirmed by HSQC, TOCSY correlations (Table 1).

### 3.2. Quantification of major *Balanites* metabolites using NMR

The absolute quantification of identified metabolites in *Balanites* parts was also estimated using ^1^H-NMR by integrating their well-determined respective peaks in the NMR spectra expressed as μg per mg dry powder (Table 2). Fatty acids amounted to a major metabolite class in all *Balanites* parts at the highest level of 134.4 μg mg^−1^ in *Balanites* seeds (BS) *versus* lowest level at *ca.* 12.5 μg mg^−1^ in the *Balanites* stem (BST), consistent with the fact of the storage of plant fatty acids in seeds.^35^ The abundance of unsaturated fatty acids in all *Balanites* parts especially seeds is suggestive of their importance as functional foods to decrease serum LDL cholesterol and the risk of atherosclerosis.^36,37^ Regarding amino acids, threonine represented the major amino acid in all parts ranging from 1.2 to 14.2 μg mg^−1^. l-Threonine is an essential amino acid known for its role in immune function regulation and improving antidiabetic activity.^38,39^ Valine and alanine were detected at lower levels ranging from 0.7 to 6.1 and 0.8 to 2.8 μg mg^−1^, respectively. Further, choline was quantified in all organ extracts, however, at low concentrations at *ca.* (0.2–1.3 μg mg^−1^). With regard to sugars, sucrose was the predominant form at 0.2 to 2.9 μg mg^−1^, whereas glucose (α- and β-forms) and galactose were detected at trace levels in all parts and suggestive that di-sugars account for *Balanites* taste.

The hypo-glycemic alkaloid, trigonelline^1^ was detected in all parts ranging from 0.2 to 1.08 μg mg^−1^, with immature fruit being the most rich. Whereas, diosgenin to represent saponins was detected at the highest level in seeds at 4.7 μg mg^−1^*versus* 0.4 μg mg^−1^ in the stem. The flavonoid isorhamnetin was detected in all parts at 0.1–1.3 μg mg^−1^, except for immature fruits.

### 3.3. Multivariate data analysis of NMR dataset

**Fig. 1:**
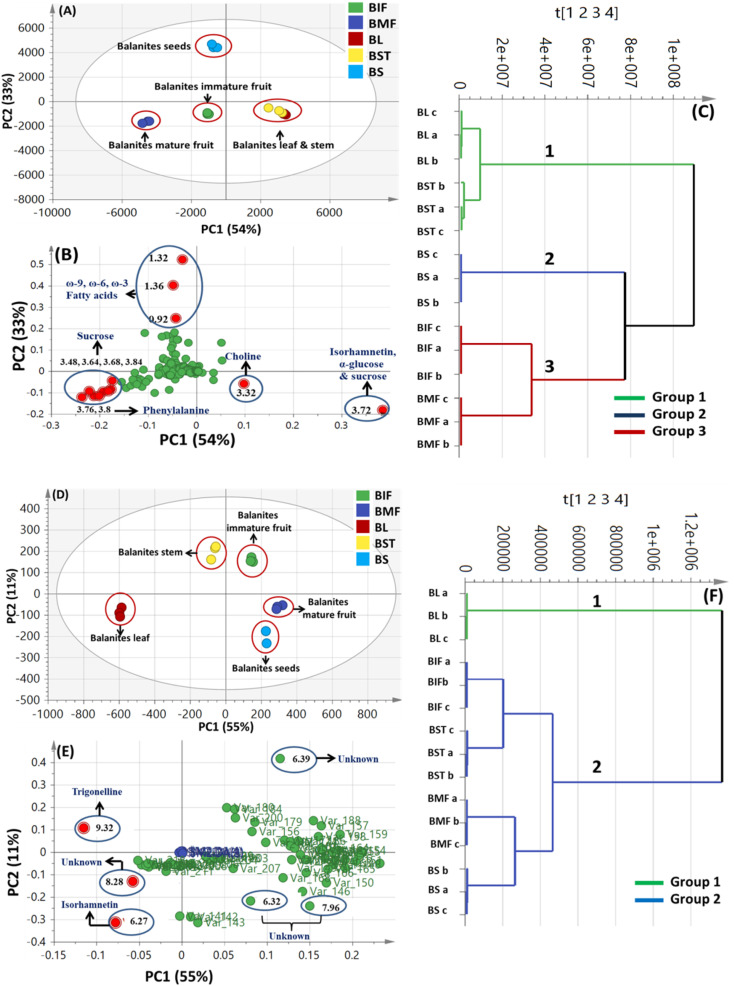
Unsupervised multivariate data analyses of *Balanites* parts dataset in the full ^1^H-NMR scale (*δ*_H_ 0–10 ppm) (A–C) and the aromatic region (*δ*_H_ 5.5–10 ppm) in (D–F). (A and D) Principal component analysis (PCA) score plot of PC1 *vs.* PC2. (B and E) Loading plot for PC1 and PC2 showing the major signals contributing to sample discrimination. (C and F) Hierarchical cluster analysis (HCA). The metabolites assignment is summarized in Table S1.† The model is colored according to marked groups.

A total of 15 samples were subjected to modelling in this study (5 different parts from the same plant; each represented by 3 biological replicates) for identification of markers among the different parts. Both PCA and HCA were initially constructed to discriminate between parts' metabolic profiles in two attempts, from all spectral width (*δ* 0.4–10.0 ppm) and from the aromatic region only (*δ* 5.5–10.0 ppm) to more focus on secondary metabolites in *Balanites* parts, *i.e.*, alkaloids, and flavonoids (Fig. 1).

#### 3.3.1. Unsupervised analyses of *Balanites* NMR dataset

The first PCA model based on the full ^1^H-NMR spectrum (*δ* 0–10 ppm) provided an overview of the primary and secondary metabolites abundance derived mostly from the aliphatic upfield (*δ* ≤ 5.4 ppm) and the aromatic downfield regions (*δ* > 5.5 ppm), respectively. Based on the full *δ*_H_ scale, PCA score plot accounted for 87% of the total variance (Fig. 1A). An obvious segregation between *Balanites* leaf and stem from other *Balanites* parts could be observed alongside PC1, with positive score values for leaf and stem samples (right side in PC1), whereas seed, mature and immature fruit samples were clustered with negative score values (left side in PC1). Further, PC2 explaining 33% of the total variance was segregated in *Balanites* seed at the upper positive side from mature and immature fruit samples at the lower negative side. Examination of the loading plot (Fig. 1B) revealed that sugars, *viz.* α-Glucose and sucrose as well as isorhamnetin flavonoid could be recognized as markers for *Balanites* fruit and stem parts, whereas unsaturated fatty acids were found to be associated with *Balanites* seeds at *δ* 1.32, 1.36 and 0.92 ppm. Similarly, the HCA dendrogram (Fig. 1C) revealed the clustering of *Balanites* parts in three distinct groups where the leaf and stem were segregated in one group, seed replicates clustered in another group besides mature and immature fruit specimens, which were likely to be comparable in composition. Likewise, to help identify variation within the aromatic region (*δ*_H_ 5.5–10.0 ppm), another PCA model (Fig. 1D) was employed. The main principal component (PC) to differentiate specimens in PCA, *i.e.* PC1 accounted for 55% of the total variance with *Balanites* leaf and stem positioned at the left side (negative PC1 score value), whereas seeds, mature and immature fruits segregated on the right positive side. The PCA loading plot (Fig. 1E) revealed trigonelline being a significant marker for the stem sample, whereas isorhamnetin was more associated with the leaf leading to its segregation on the far-left lower quadrant negative PC1 and PC2. Unknown signals (*δ* 6.39, 6.32, and 7.96 ppm) appeared to discriminate seeds, and mature and immature fruits that are yet to be identified. HCA (Fig. 1F) was segregated into two groups in samples; one for leaf samples whereas the other parts *i.e.*, stem, seed, mature, and immature fruits clustered in another group.

#### 3.3.2. Supervised OPLS-DA analysis of *Balanites* NMR dataset

The supervised orthogonal partial least square discriminant analysis (OPLS-DA) was further employed to aid in sample discrimination that failed during unsupervised analysis, *i.e.*, mature fruits against immature ones. OPLS-DA model was performed considering only the aromatic region (*δ* 5.5–10.0 ppm), where mature fruit was modeled against immature one (Fig. S2A†).

OPLS model exhibited *Q*^2^ = 0.97, indicating strong model predictability and total variance coverage of 99% (*R*^2^ = 0.99). The respective loading *S*-plot (Fig. S2B†) revealed that trigonelline was the major distinctive metabolite in immature fruit, whereas isorhamnetin was the major distinct marker in the mature fruit sample, though with a non-significant *p*-value of 0.18.

### 3.4. Metabolites profiling of *Balanites via* UHPLC-ESI-QTOF-MS/MS

**Fig. 2:**
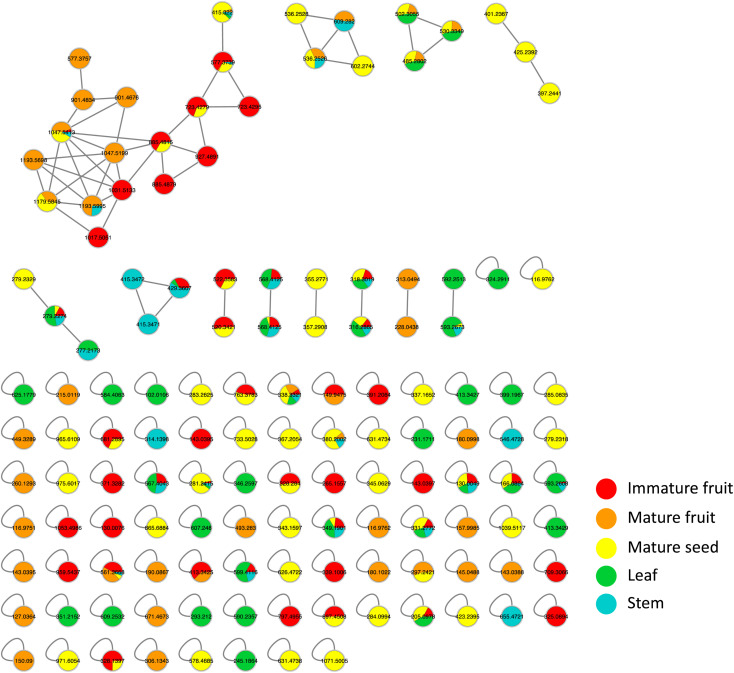
A whole molecular network of MS/MS data of metabolites detected in *Balanites aegyptiaca* different parts in positive ionization mode. Nodes are labeled with parent mass. The network is displayed as pie chart with different colors representing relative distribution of metabolites based on feature peak areas of in the different parts.

Metabolites profiling of the different *B. aegyptiaca* parts was carried out *via* UHPLC-ESI-QTOF-MS/MS (Fig. S3†), in an attempt to identify the distribution of secondary metabolites. Data were further processed employing the feature-based molecular networking (FBMN) approach and web-based tool GNPS. The output of data processing using MZmine 2 included a total of 133 features after removing features corresponding to methanol blank, in-source fragments, and sodium adducts. GNPS-FBMN workflow was performed generating a molecular network where structurally-related metabolites were linked together based on the similarity of their MS/MS fragmentation. The generated molecular network (Fig. 2) showed 45 features comprising 12 spectral families in addition to 88 singleton features. As listed in Table 3, 39 metabolites were identified belonging to different chemical classes including steroidal saponins, oxylipids, phenolic amides, and flavonoids. Visual analysis of MS/MS data *via* molecular networking enabled the annotation of metabolites, together with highlighting differential features between dissimilar samples that are different parts of *B. aegyptiaca*. The node pie chart color reflected the relative abundance of features in different parts.

**Table 3:** Identified steroidal saponin metabolites in the positive mode of *Balanites aegyptiaca* different parts using UHPLC-MS/MS

No.	*R*_t_ (min)	Compound name	*m*/*z*	Molecular formula	Mass error (ppm)	MS^2^ product ions	IF^a^	MF^a^	MS^a^	L^a^	ST^a^
**Steroidal saponins**											
1	6.58	Diosgenin	415.322	C_27_H_42_O_3_^+^	3.2	397.3114, 379.2969, 271.2062, 253.1950	—	√	√	√	√
2	5.49	Diosgenin-26-hexoside	577.3757	C_33_H_53_O_8_^+^	3.8	415.3216, 271.2064, 253.1959	—	√	—	—	—
3	6.58	Diosgenin-3-hexoside	577.3739	C_33_H_53_O_8_^+^	0.7	433.2453, 271.1976, 253.1870	√	√	√	—	√
4	6.59	Diosgenin hd	723.4279	C_39_H_63_O_12_^+^	3.3	579.3007, 577.3590, 433.2454, 415.3092, 397.2987, 271.1978, 253.1870	√	—	√	—	—
5	7.34	Diosgenin hd isomer	723.4295	C_39_H_63_O_12_^+^	−2.6	579.3166, 4 332 488, 415.3213, 271.2047, 253.1945	√	—	—	—	—
6	6.47	Diosgenin hdh	885.4879	C_45_H_73_O_17_^+^	4.1	741.3710, 739.4310, 723.4351, 577.3663, 415.3200, 309.1176, 271.2092, 253.1960	√	—	—	—	—
7	6.80	Diosgenin hdh	885.4815	C_45_H_73_O_17_^+^	−3.1	741.3469, 723.4102, 577.3587, 415.3086, 309.1081, 271.1972, 253.1871	√	—	√	—	—
8	7.25	Diosgenin hdha	927.4891	C_47_H_75_O_18_^+^	−6.1	783.3533, 781.4193, 577.3549, 415.3093, 351.1160, 271.1974, 253.1870	√	—	—	—	—
9	3.92	Diosgenin hhh	901.4834	C_45_H_73_O_18_^+^	4.7	739.4308, 595.3138, 577.3756, 433.2610, 415.3220, 271.2064, 253.1967	—	√	—	—	—
10	4.68	Diosgenin hhh isomer	901.4824	C_45_H_73_O_18_^+^	3.6	739.4279, 577.3749, 415.3217, 271.2061, 253.1952	—	√	—	—	—
11	6.55	Diosgenin hdhd	1031.5432	C_51_H_83_O_21_^+^	1.0	885.4841, 723.4326, 577.3740, 415.3215	√	—	—	—	—
12	6.59	Diosgenin hdhp	1017.5322	C_50_H_81_O_21_^+^	5.6	871.4772, 723.4346, 577.3757, 415.3224	√	—	—	—	—
13	3.87	Diosgenin hdhh	1047.5413	C_51_H_83_O_22_^+^	4.0	885.4874, 723.4327, 577.3743, 415.3219, 271.2061, 253.1966	√	√	√	—	√
14	3.85	Diosgenin hdhhp	1179.5845	C_56_H_90_O_26_^+^	4.4	1017.5343, 885.4865, 723.4343, 577.3751, 415.3216, 271.2047	—	√	√	—	—
15	3.83	Diosgenin hdhhd	1193.5995	C_57_H_93_O_26_^+^	3.8	1031.5431, 885.4876, 723.4333, 577.3747, 415.3214, 271.2057, 253.1945	—	√	—	—	√
16	4.58	Diosgenin hdhhd isomer	1193.5615		High mass error		—	√	—	—	—

**N-Containing metabolites**											
17	0.82	Xanthosine	285.0835	C_10_H_13_N_4_O_6_^+^	1.9	153.0406, 133.0492, 115.0385	—	—	√	—	—
18	1.51	Tryptophan	205.0978	C_11_H_13_N_2_O_2_^+^	3.1	188.0708, 146.0603, 118.0654	√	—	√	√	—
19	11.75	Pheophorbide A	593.2608	C_39_H_37_N_4_O_5_^+^	High mass error	533.2387	√	—	—	√	√
20	12.09	Pheophorbide A isomer	593.2778	C_39_H_37_N_4_O_5_^+^	3.3	533.2563	—	—	√	√	√

**Phenolics**											
21	3.14	Narcissin	625.1779	C_28_H_33_O_16_^+^	2.5	479.1154, 317.0659, 129.0546					
22	3.98	Feruloyltyramine	314.1398	C_18_H_20_NO_4_^+^	3.5	177.0542, 145.0279, 121.0648					

**Fatty acids and lipids**											
23	12.88	Sphingolipid	568.4125	C_39_H_59_NO_5_P^+^	−0.07	476.3528, 430.3099, 338.2504	√	√	√	√	√
24	13.49	Sphingolipid	568.4125	C_39_H_59_NO_5_P^+^	−0.07	476.3512, 430.3104, 338.2507	√	—	—	√	√
25	0.84	2-Methylideneglutaric acid	145.0488	C_6_H_9_O_4_^+^	−5.1	127.0389, 104.0082	—	√	—	—	—
26	7.79	Stearidonic acid	277.2173	C_18_H_29_O_2_^+^	3.9	235.1691, 221.1574, 207.1386, 149.1319, 135.1168, 121.1007	—	—	—	√	√
27	9.34	Octadecatrienoic acid	279.2318	C_18_H_31_O_2_^+^	−0.2	261.2231, 243.2109, 165.1266, 147.1159	—	—	√	—	—
28	8.43	Octadecatrienoic acid isomer	279.2318	C_18_H_31_O_2_^+^	−0.2	250.4211, 243.2113, 218.9202, 173.1314, 135.1160, 109.1009	—	—	√	—	—
29	12.05	Octadecenoic acid	283.2625	C_18_H_35_O_2_^+^	−2.3	247.2463, 201.2660, 163.1493, 149.1329, 135.1170, 109.1023	—	—	√	—	—
30	6.05	Dehydrophytosphingosine	316.2865	C_18_H_38_NO_3_^+^	5.9	298.2716, 280.2650, 250.2534, 147.1166, 119.0840	√	—	√	√	√
31	6.33	Phytosphingosine	318.3019	C_18_H_40_NO_3_^+^	5.1	300.2909, 282.2797, 264.2682, 252.2690	√	—	√	√	√
32	9.49	Anandamide	324.2911	C_20_H_38_NO_2_^+^	4.3	306.2844, 227.8998	—	—	—	√	—
33	6.53	*N*-Palmitoyl alanine	328.2840	C_19_H_38_NO_3_	−1.9	310.2755, 298.2737, 280.2647, 263.2365, 242.9088, 195.1409, 151.1485	√	—	√	—	—
34	3.93	Octacosahexaenoic acid	413.3425	C_28_H_45_O_2_^+^	1.9	395.3305, 217.1591, 199.1485	√	√	—	—	—
35	4.83	Octacosahexaenoic acid isomer	413.3427	C_28_H_45_O_2_^+^	3.1	395.3325, 327.2679	—	—	—	√	—
36	4.57	Octacosahexaenoic acid isomer	413.3429	C_28_H_45_O_2_^+^	3.6	395.3309, 217.1581, 199.1487	—	—	—	√	—
37	7.57	Phospahtidyl choline (18 : 2/0 : 0)	520.3421	C_26_H_51_NO_7_P^+^	4.5	502.3300, 184.0732, 104.1069	√	—	√	—	—
38	8.24	Phospahtidyl choline (0 : 0/18 : 1)	522.3583	C_26_H_53_NO_7_P^+^	5.5	504.3471, 184.0735, 104.1071	√	—	√	—	—
39	13.10	2-Deoxyecdysone	449.3289	C_27_H_45_O_5_^+^	6.1	431.3203, 253.1142, 187.0693	—	√	—	—	—

a IF: immature fruit; MF: mature fruit; MS: mature seed; L: leaf; ST: stem.

#### 3.4.1 Identification of steroidal saponins

**Fig. 3:**
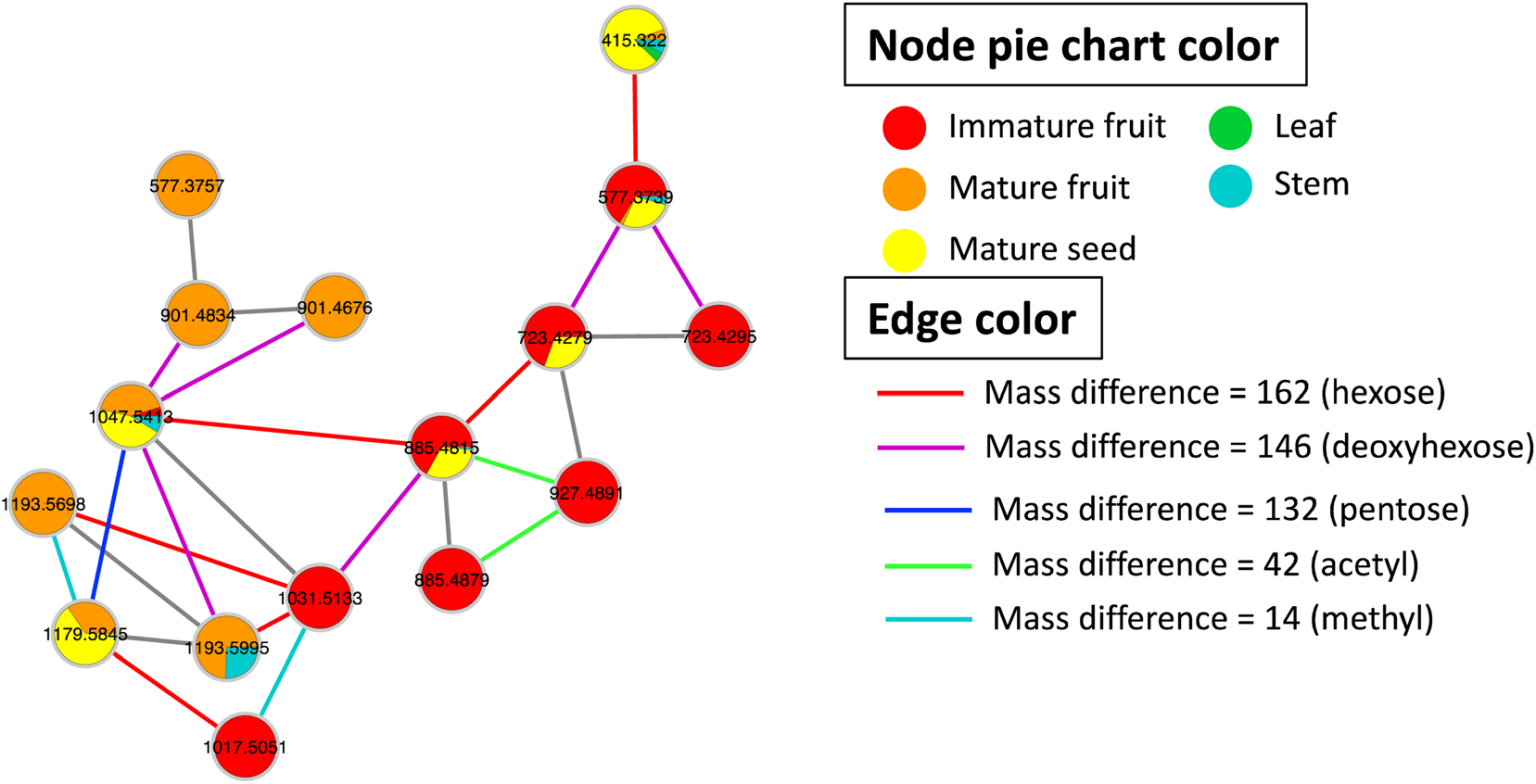
GNPS molecular network of steroidal saponins detected in *Balanites aegyptiaca* different parts. Nodes are labeled with precursor mass. Each node is displayed as pie chart with different colors representing relative distribution of metabolites based on feature peak areas in different parts. Edge color represents the mass difference between nodes.

*B. aegyptiaca* is known to be rich in steroidal saponin glycosides. The MS/MS-based molecular network revealed a spectral family of 16 metabolites belonging to the steroidal saponin class (Fig. S4†). The tentative identification of these metabolites was assisted by characteristic neutral losses due to the fission of the aglycone and the sugar moieties obtained by tandem MS. In addition, two hits resulting from the GNPS library search for *m*/*z* 415.322 [M + H]^+^ and *m*/*z* 1047.5413 [M + H]^+^ were assigned as diosgenin aglycone and diosgenin tetrasaccharide, respectively. The first identified metabolite *m*/*z* 415.322 [M + H]^+^ C_27_H_42_O_3_ was assigned as diosgenin aglycone based on the MS/MS data (Fig. S4b†) showing two characteristic product ions at *m*/*z* 271.2062 and *m*/*z* 253.1950, corresponding to the subsequent neutral losses of C_8_H_16_O_2_ and H_2_O due to fission of bond C17–C20 and elimination of H_2_O, respectively (Fig. S4a†). In addition to diosgenin aglycone, two isomeric peaks with [M + H]^+^ at 577.3757 and 577.3739 were eluted at different RTs 5.49 and 6.58 min, respectively. The mass difference from diosgenin aglycone at 162 amu corresponded to an additional hexose moiety that can be attached at either C3 of the spirostanol skeleton or C26 of the furostanol skeleton. In MS/MS fragmentation, the former isomer showed prominent product ion at *m*/*z* 415.3217 corresponding to the direct loss of hexosyl moiety followed by fission of aglycone to yield diagnostic ions indicating the glycosylation at C26. Whereas, MSMS fragmentation of the second isomer eluting at RT 6.58 min revealed hexosyl moiety being connected at the C3 position, as no obvious product ion at *m*/*z* 433.2584, resulting from the fission of aglycone followed by the elimination of hexosyl to form a diagnostic ion at *m*/*z* 271.2062 was noted, (Fig. S5 and S6†). Interestingly, the distribution of the two isomers was different among *Balanites* parts, where furostanol was present in mature fruit only, while spirostanol isomer was predominant in immature fruit followed by mature seeds (Fig. S5†), suggestive of different steroid biosynthetic pathways in parts. The fragmentation pattern includes the neutral loss of C_8_H_16_O_2_ directly from the precursor ion occurring in spirostanol saponins, while the elimination of sugar moieties from precursor ions yields furostanol isomers. The detailed annotated steroidal saponin glycosides are listed in Table 3, Fig. 3, S5 and S6.†

#### 3.4.2. Fatty acids and lipids

Next to saponins, fatty acids, and lipids were detected as the second abundant class in *Balanites* represented by 17 peaks and predominated by saturated, unsaturated, and hydroxylated forms. Among identified lipids, peak 23 (*m*/*z* 568.4125, C_32_H_59_NO_5_P^+^), peak 30 (*m*/*z* 316.2865, C_18_H_38_NO_3_^+^), and peak 31 (*m*/*z* 318.3019, C_18_H_40_NO_3_^+^) were commonly identified in *Balanites* parts and annotated as sphingolipid, dehydrophytosphingosine, and phytosphingosine, respectively. Moreover, 3 unsaturated fatty acids were exclusively identified in *Balanites* mature seeds, including octadecatrienoic acid (γ-linolenic acid) (279.2318, C_18_H_31_O_2_^+^), octadecatrienoic acid isomer (279.2318, C_18_H_31_O_2_^+^), and octadecenoic acid (oleic acid) (283.2625, C_18_H_35_O_2_^+^). γ-Linolenic acid and oleic acid are essential fatty acids with several health benefits such as anti-inflammatory and lipid-lowering action.^40^ Two nitrogenous lipids manifested in peaks 33 and 37 were detected only in immature fruit and seed, annotated as *N*-palmitoyl alanine (328.2840, C_19_H_38_NO_3_), phosphatidyl choline (18 : 2/0 : 0) (520.3421, C_26_H_51_NO_7_P^+^), and phosphatidyl choline (0 : 0/18 : 1) (522.3583, C_26_H_53_NO_7_P^+^). Phosphatidyl choline is a glycerophospholipid with anti-inflammatory action in arthritis and ulcerative colitis^41^ and supports the health benefits of immature fruit and seeds. Phosphatidylcholine (PC) is an important cell membrane component that is critical for cell structure and membrane stability maintenance, in addition to being a plant growth regulator.^42^ Phosphatidylcholine application was also found to enhance homeostasis against salt stress.^43^ In contrast, few reports are found in the literature on lysophospholipids except for their role as priming agents of the plant immune system and resistance against pathogens.^44^ A detailed comparison between phospholipids and lysophospholipids regarding plants' effects is yet to be reported.

#### 3.4.3 Phenolics and nitrogenous compounds

Compared to saponins and fatty acids, only two phenolics were detected (peak 21 and 22) annotated as narcissin (625.1779, C_28_H_33_O_16_^+^) and feruloyltyramine (314.1398, C_18_H_20_NO_4_^+^), respectively. Narcissin(isorhamnetin-3-*O*-rutinoside), a flavonoid with potential health benefits *viz*. enhances coronary blood flow, anticancer, anthelmintic, and antiprotozoal^45^ was the major flavonoid. Likewise, N-containing metabolites were detected though at lower levels, among which tryptophan (peak 18) (205.0978, C_11_H_13_N_2_O_2_^+^) was detected in three parts *viz.* immature fruits, seed, and leaf. Pheophorbide A (593.2608, C_35_H_37_N_4_O_5_^+^) and its isomer, chlorophyll derivatives with potential anticancer activity^46^ were detected in immature fruits, leaf, and stem.

### 3.5 Multivariate data analysis of *Balanites* UPLC-MS dataset

#### 3.5.1 Unsupervised multivariate data analysis of *Balanites* UPLC-MS dataset

**Fig. 4:**
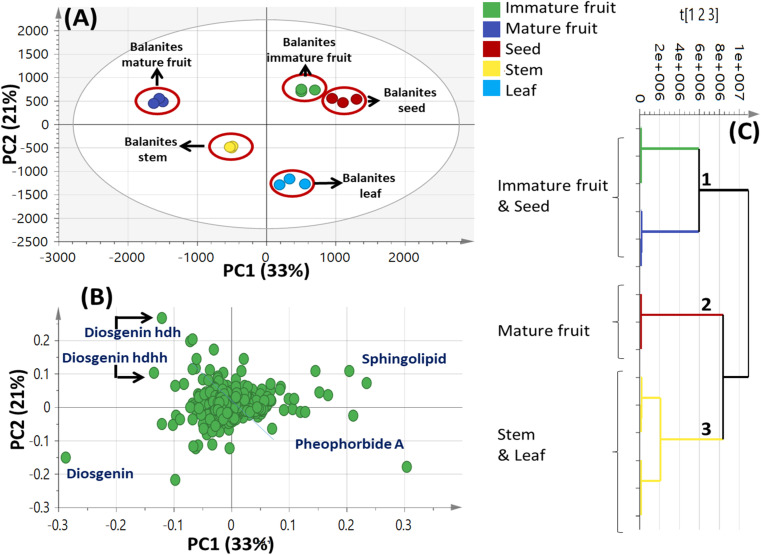
Unsupervised multivariate data analyses of *Balanites* parts metabolite profiles *via* UPLC-MS. (A) Principal component analysis (PCA) score plot of PC1 *vs.* PC2. (B) Loading plot for PC1 and PC2 showing mass peaks and their assignments. (C) Hierarchical cluster analysis (HCA). The metabolites assignment is summarized in Table 2. The model is colored according to marked groups.

Although differences in metabolites profile were observed, PCA and HCA were attempted as more holistic approaches to test for heterogeneities among the different parts of *B. aegyptiaca*. The UPLC-MS-based PCA metabolome clusters were located at different points in the two-dimensional space prescribed by two vectors, that is PC1 and PC2 to account for 33% and 21%, respectively, of the variance showing obvious segregation between *Balanites* mature fruit and stem from other *Balanites* parts along PC1, with mature fruit and stem samples positioned with negative score values (left side in PC1), whereas seed, leaf and immature fruit samples clustered to the right side of PC1, (Fig. 4A). Examination of the loading plot (Fig. 4B) revealed that lipids, *viz.* sphingolipid as well as a nitrogenous metabolite, pheophorbide A could be recognized as markers for *Balanites* immature fruit, seed and leaf parts, whereas steroidal saponins, *viz.* diosgenin hdh and diosgenin hdhh were found to be associated with *Balanites* mature fruit, and suggestive that fruit is the richest in diosgenin conjugates. Similarly, the HCA dendrogram (Fig. 4C) revealed the clustering of *Balanites* parts in three distinct groups, where immature fruit and seed were segregated in one group, mature fruit clustered in another group besides stem and leaf specimens, which were likely to be comparable were grouped together.

#### 3.5.2 Supervised OPLS-DA analysis of *Balanites* UPLC-MS dataset

**Fig. 5:**
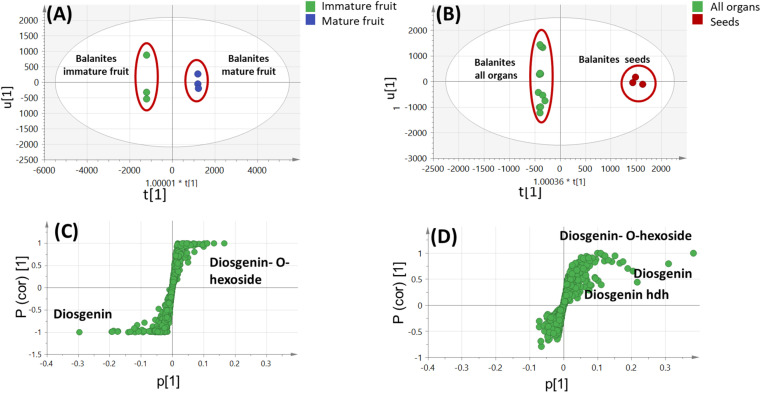
UPLC-MS-based OPLS-DA score plot (A) derived from modeling *Balanites aegyptiaca* immature fruit *versus* mature fruit (*n* = 3). (B) Derived from modeling *B. aegyptiaca* seeds *versus* other 4 parts (*n* = 3). (C) and (D) The respective loading *S*-plots showing the covariance p [1] against the correlation p (cor) [1] of the variables of the discriminating component of the OPLS-DA model. Cut-off values of *p* < 4.99962e−005 was used. Designated variables are highlighted and identifications are discussed in the text.

Supervised OPLS-DA was attempted to build a better classification model constructed with *Balanites* immature fruit modelled against mature ones (Fig. 5A). OPLS-DA has a great capability for marker metabolites discrimination by assignment of the most relevant variables among two classes.^47^ OPLS model exhibited *Q*^2^ = 0.99 indicating model predictability, and total variance coverage of 99% (*R*^2^ = 0.99). The respective loading *S*-plot (Fig. 5B) revealed that diosgenin was a major distinctive metabolite in immature fruit, whereas diosgenin-*O*-hexoside was the major steroid in mature fruit sample, with a *p*-value of 80%, and suggestive of activation of specific glycosyl transferase upon fruit maturation to yield diosgenin saponins. OPLS-DA supervised modeling of seeds against all other parts (Fig. 5C) was further attempted for better segregation. The observed segregation in the score plot (Fig. 5D) was attributed to seed enrichment in diosgenin-*O*-hexoside, diosgenin, and diosgenin hdh. OPLS model validations showed low residual SECV for the OPLS model, high variance, and prediction coverages as represented by *R*^2^ and *Q*^2^ values, Fig. S7.†

### 3.6. GC-MS profiling of metabolites in *B. aegyptiaca* extract

**Fig. 6:**
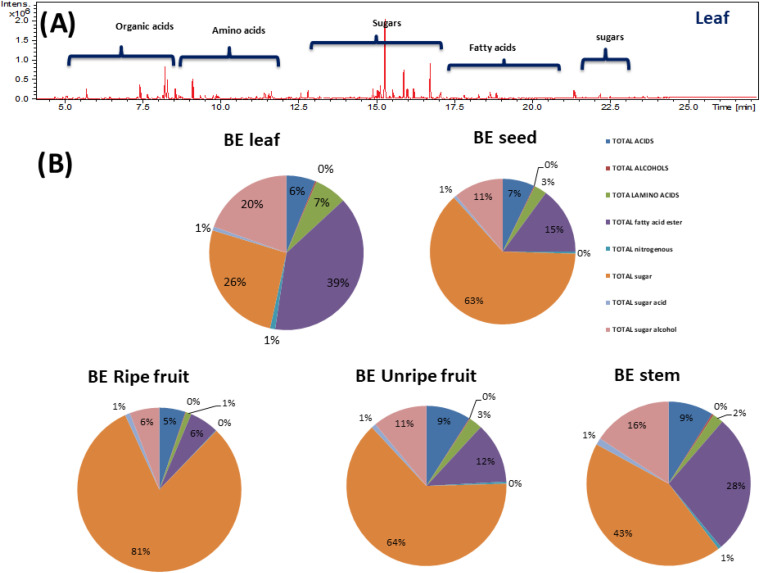
(A) Representative GC-MS chromatograms of TMS derivatives of primary metabolites from *Balanites aegyptiaca* different parts. (B) Pie chart of the major groups of primary metabolites identified from *Balanites aegyptiaca* in different parts.

Although NMR provided insight into the major primary metabolites, GC-MS is more sensitive for the identification of less abundant metabolites not detected using NMR. GC-MS post-silylation analysis was employed to provide a holistic profile of primary metabolites from *B. aegyptiaca* parts. A total of 135 metabolites (Table 3 and Fig. 6A) were identified belonging to sugars (mono- and disaccharides), sugar alcohols, sugar acid, amino acids, fatty acids, esters, organic acids, and alcohols, in addition to a few nitrogenous compounds (Fig. 6B).

#### 3.6.1 Sugars

Sugars were detected as the most dominant primary metabolites in *B. aegyptiaca* parts represented by 41 peaks, which amounted to a total of 391, 205, 139, 68, and 44 mg g^−1^ in ripe fruits, unripe fruits, seeds, stem, and leaves, respectively. Both ripe and unripe fruits were found the richest in sugars, which makes them edible and palatable for humans. β-d-Glucopyranose (peaks 106) was detected as the major sugar in all parts, detected at the highest levels in ripe fruit (102.4 mg g^−1^) followed by unripe fruit (33.7 mg g^−1^), whereas ranging at 10.0–27.9 mg g^−1^ in other parts. Next to glucose, mannose (peak 103) was identified as the second major sugar in both ripe and unripe fruits amounting to 93.5 and 26.6 mg g^−1^, respectively. Fructose, a chief dietary sugar with high relative sweetness and a lower effect on blood insulin level^48^ was detected at the highest level in ripe fruit at 95.7 mg g^−1^. Interestingly, co-administration of fructose with glucose can increase intestinal absorption of fructose,^48^ suggestive that *B. aegyptiaca* ripe fruits with higher content of glucose and fructose are beneficial as a dietary supplement for diabetic patients. Additionally, tagatofuranose (peak 96) was detected in ripe fruits at 52.5 mg g^−1^ and at much lower levels in all other parts. Sucrose, the major disaccharide was detected at the highest levels in unripe fruits, seeds, and ripe fruit at 113.8, 54.9, and 24.5 mg g^−1^, respectively.

Sugar alcohols were detected at relatively high levels in *B. aegyptiaca* parts ranging from 24.9 to 35.6 mg g^−1^ with the highest level in unripe fruits (35.6 mg g^−1^) followed by leaves and ripe fruits at *ca*. 30 mg g^−1^. Sugar alcohol is a low-calorie sweetener without any effect on blood glucose level as they are not absorbed from the intestine and is widely used as a sweetener for diabetic patients.^49^d-Pinitol (peak 129) was detected as the major sugar alcohol in *B. aegyptiaca* parts at the highest level in the unripe fruits at 28.96 mg g^−1^ followed by leaves at 26.1 mg g^−1^ posing unripe fruit as low-calorie source sugar. d-Pinitol, a polyol derivative is reported for its antidiabetic properties as it exerts insulin-like hypoglycemic effect^50^ and is likely to augment trigonelline antidiabetic action in *Balanites*.

Moreover, myo-inositol (peaks 133 and 135), an insulin sensitizer sugar alcohol was likewise detected in parts though at much lower levels *ca*. 1.3–2.9 mg g^−1^. Glycerol, a simple polyol with applications in pharmaceutical, cosmetic, and food industries^51^ was detected (peak 126) at considerable levels in ripe fruits and leaves at 4–5 mg g^−1^ and accounting for fruit sweetness. Such evidence-based results provide new insight into *B. aegyptiaca* fruit as antidiabetics.

Sugar acids were detected in all *B. aegyptiaca* parts ranging from 1 to 5 with higher abundance in ripe fruits at 4.7 mg g^−1^. Ribonic acid (peak 121) and glucaric acid (peaks 123 and 124) were the major sugar acids. d-Glucaric acid is used as a food additive as well as in the pharmaceutical industry and dietary supplements.^52^

#### 3.6.2. Organic acids/alcohols/inorganics

Organic acids play a role in fruit's taste and act as a natural in addition to improving digestion.^53^ Organic acids were detected in *B. aegyptiaca* parts, with the highest level found in unripe fruits (29.1 mg g^−1^) followed by ripe fruits (24.9 mg g^−1^) *versus* lowest in leaves (10.2 mg g^−1^) represented by malic and glyceric acids. Malic acid is a well-known antioxidant and preservative. Glyceric acid (peak 11) was detected at higher levels in vegetative parts (leaves and stem) at *ca*. 6 mg g^−1^, compared to malic acid found more abundant in fruits. The amount of organic acids is an important indicator of edible fruit quality.^53^ Another metabolite with potential preservative action is phosphoric acid (peak 9) detected at a much higher level in fruits and seeds at 7–10 mg g^−1^. Alcohols were detected at trace levels in all parts ranging from 0.41 to 0.58 mg g^−1^ and represented mainly by 1,3 propandiol (peak 18) and nonanol (peak 20).

#### 3.6.3. Amino acids

Amino acids are considered the structural and functional units of protein formation and play a pivotal role in human body growth and physiology.^54^ Free amino acids were detected at comparable levels in all parts represented by 31 peaks ranging from 3.4 to 11.4 mg g^−1^, with the highest levels in leaves followed by unripe fruits. The major amino acids included alanine (peak 22), valine (peak 29), l-leucine (peak 31), and tyrosine (peak 52).

#### 3.6.4. Nitrogenous compounds

In addition to amino acids, nitrogenous compounds were detected at comparable levels in all parts represented by γ-aminobutyric acid (peak 72), nicotinic acid (peak 67), and trigonelline (peak 71). γ-Aminobutyric acid (peak 72), is an important neurotransmitter that plays a pivotal role in brain health (Baky *et al.*, 2022), whereas nicotinic acid (peak 67) and trigonelline (peak 71) are structurally similar compounds reported for their hypoglycemic action and to contribute for fruits antidiabetic effect.^55^ From the aforementioned findings, *B. aegyptiaca* could be used as a nutritional supplement for diabetic patients owing to its richness in sugar alcohols and trigonelline.

#### 3.6.5. Fatty acids/esters/sterols

Fatty acids/esters were detected at relatively high levels in *B. aegyptiaca* with the highest in green leaf and stem parts at 65.99 and 43.71 mg g^−1^, respectively. Fatty acids profile revealed peaks belonging to saturated fatty acids (SFA), mono-unsaturated fatty acids (MUFA), and poly-unsaturated fatty acids (PUFA). SFA was most abundant represented by heneicosanoic acid (peak 62), 10.24–15.09 mg g^−1^ followed by palmitic (peak 56) and stearic acid (peak 60) detected at 7–13 mg g^−1^. The absence of SFAs from edible fruit parts proves that they are healthier than vegetative parts. Regarding unsaturated fatty acids, oleic acid (peak 58), a MUFA with hypocholesterolemic effect^8^ was detected in *B. aegyptiaca* parts at 1.6–4.5 mg g^−1^. α-Linolenic (ALA) (peak 59) was the major PUFA detected in *B. aegyptiaca* parts at 10.5 mg g^−1^ in the leaves *versus* the lowest level in ripe fruits at 0.67 mg g^−1^. Likewise, linoleic acid (peak 57) another PUFA was detected in *B. aegyptiaca* parts ranging from 1 to 5 mg g^−1^ with the highest levels found in leaves. Regarding fatty acids acyl ester, 1-monopalmitin (peak 63) was detected as the major fatty acid ester in all *B. aegyptiaca* parts ranging from 1.13 and 3.51 mg g^−1^. In general, and compared to fruit's richness in sugars, leaves appeared to be most rich in fatty acids.

### 3.7. Multivariate data analysis of *Balanites* GC-MS dataset

#### 3.7.1. Unsupervised multivariate data analysis of *Balanites* GC-MS dataset

**Fig. 7:**
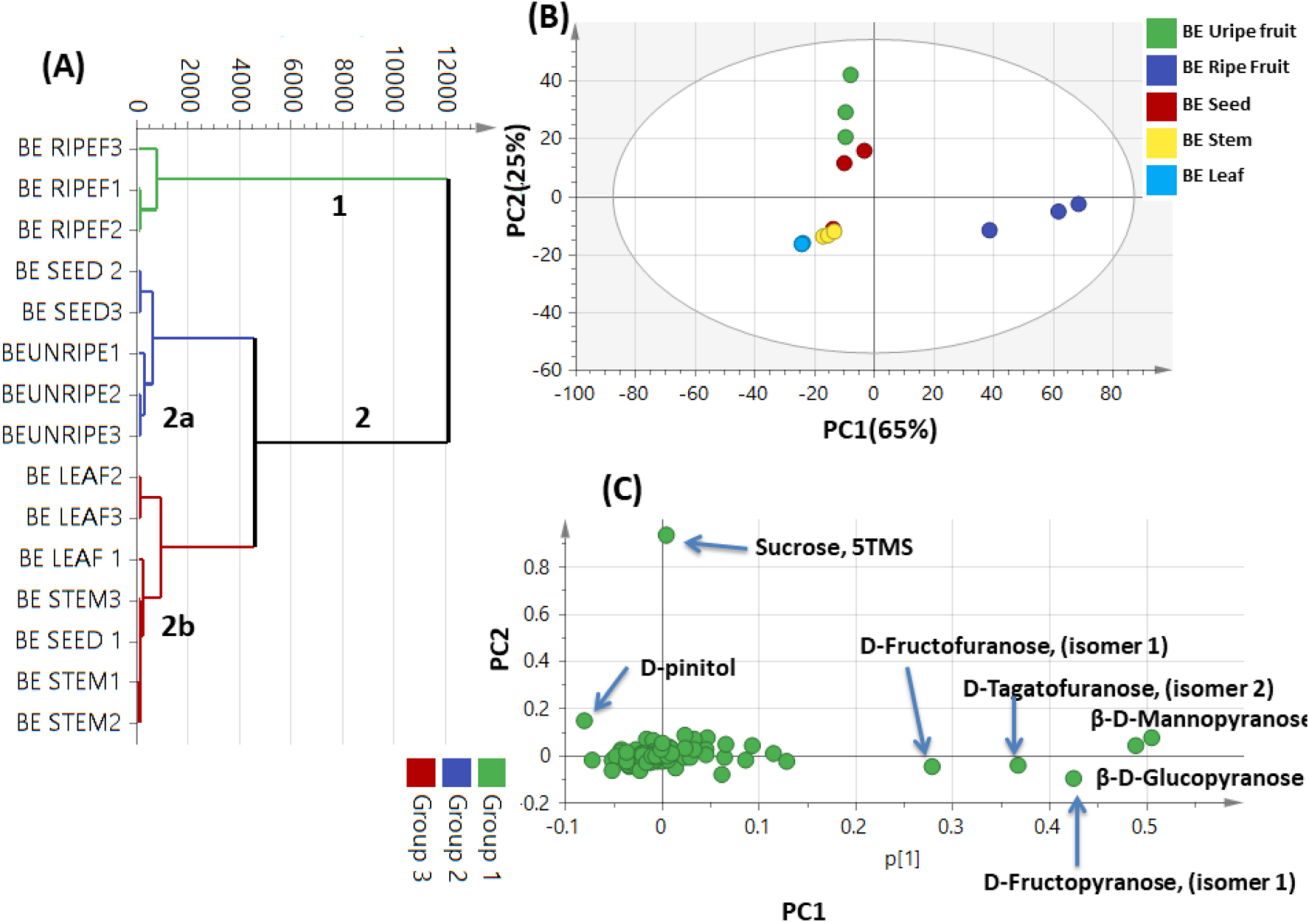
Unsupervised multivariate data analyses of the studied parts of *Balanites aegyptiaca* derived from modeling silylated primary metabolites analyzed *via* GC-MS (*n* = 3). (A) HCA plot. (B) PCA score plot of PC1 *vs.* PC2 scores. (C) The respective loading plot for PC1 and PC2, providing mass peaks and their assignments. The metabolome clusters are placed in two-dimensional space at the distinct locations defined by two vectors of principal component PC1 = 65% and PC2 = 25%.

Primary metabolites heterogeneity among *B. aegyptiaca* parts was further assessed using multivariate data analyses including HCA and PCA modelling of the GC-MS dataset (Table 4). HCA depicted a dendrogram of three distinct clusters (Fig. 7A), with ripe fruits clustered separately in cluster 1, while other parts were divided into two subdivisions from cluster 2. The seeds and unripe fruits were clustered together in sub-cluster 2a, while the leaves and stem were clustered in sub-group 2b. However, clustering of the 4 parts, *i.e.*, seeds, unripe fruits, leaves, and stems together in group 2 revealed that HCA is inefficient to assess variations among parts regarding their primary metabolites profile. The unsupervised PCA model (Fig. 7B) accounted for 90% of the total variance, with clear discrimination of ripe fruits to the right side of PC1, whereas all other 4 parts were segregated at the left side of PC1 represented by two groups; one for unripe fruits and seeds at the positive side and the other leaves and stem at the negative left side. The corresponding loading plot in (Fig. 7C) revealed the relative abundance of monosaccharides in ripe fruits represented by glucose (peaks 106), mannose (peak 103), fructose (peak 97), and tagatose (peak 96) and to account for its segregation. Moreover, unripe fruits were characterized by enriched sucrose levels (peak 110) and accounting for their segregation. Additionally, d-pinitol (peak 129), was found most rich in unripe fruits.

**Table 4:** Quantitative analysis of silylated primary metabolites (mg g^−1^) in different parts of *Balanites aegyptiaca via* GC-MS, *n* = 3

Peak no.	Average *R*_t_	Average RI	Metabolite name	Class	Leaf	Ripe fruit	Seed	Stem	Unripe fruit
1	5.132	1061.73	Lactic acid, (2TMS)	Acid	0.95 ± 0.06	1.47 ± 0.08	0.94 ± 0.14	1.10 ± 0.52	2.85 ± 0.74
2	5.34	1074.65	Glycolic acid, (2TMS)	Acid	0.20 ± 0.03	0.88 ± 0.10	0.29 ± 0.04	0.24 ± 0.04	1.37 ± 0.59
3	5.528	1086.36	Pyruvic acid, (2TMS)	Acid	0.49 ± 0.04	0.05 ± 0.05	0.16 ± 0.05	0.44 ± 0.14	0.21 ± 0.09
4	6.366	1138.42	β-Lactic acid, (2TMS)	Acid	0.02 ± 0.00	0.03 ± 0.00	0.02 ± 0.00	0.02 ± 0.00	0.03 ± 0.00
5	6.591	1152.82	3-Hydroxybutyric acid, (2TMS)	Acid	0.03 ± 0.00	0.22 ± 0.06	0.04 ± 0.01	0.02 ± 0.00	0.04 ± 0.00
6	7.269	1194.52	Malonic acid, (2TMS)	Acid	0.03 ± 0.00	0.69 ± 0.10	0.18 ± 0.07	0.03 ± 0.00	0.25 ± 0.07
7	7.744	1227.75	2-Hydroxyisocaproic acid, (2TMS)	Acid	0.03 ± 0.01	0.09 ± 0.02	0.01 ± 0.01	0.01 ± 0.00	0.04 ± 0.01
8	8.236	1262.75	Itaconic acid, (2TMS)	Acid	0.22 ± 0.04	0.03 ± 0.00	0.06 ± 0.03	0.04 ± 0.00	0.11 ± 0.01
9	8.333	1270.13	Phosphoric acid, (3TMS)	Acid	0.15 ± 0.03	9.83 ± 3.73	7.26 ± 1.71	0.93 ± 0.12	9.05 ± 1.11
10	8.808	1304.17	Succinic acid, (2TMS)	Acid	0.83 ± 0.16	1.20 ± 0.34	0.53 ± 0.05	0.98 ± 0.22	1.37 ± 0.75
11	9.142	1328.09	Glyceric acid, (3TMS)	Acid	5.86 ± 0.69	0.43 ± 0.34	1.58 ± 0.30	6.27 ± 2.05	1.96 ± 1.06
12	9.248	1335.65	γ-Hydroxybutyric acid, (2TMS)	Acid	0.01 ± 0.00	0.06 ± 0.02	0.04 ± 0.00	0.02 ± 0.00	0.14 ± 0.09
13	10.244	1408.52	3-Deoxytetronic acid, (tri-TMS)	Acid	0.02 ± 0.00	0.03 ± 0.02	0.01 ± 0.01	0.01 ± 0.01	0.11 ± 0.04
14	10.742	1448.23	4-Hydroxyvaleric acid, (2TMS)	Acid	0.08 ± 0.01	0.07 ± 0.00	0.07 ± 0.00	0.07 ± 0.01	0.08 ± 0.01
15	10.868	1458.31	Ketomalonic acid hydrate,(TMS)	Acid	0.07 ± 0.02	0.02 ± 0.03	0.03 ± 0.01	0.09 ± 0.03	0.07 ± 0.05
16	11.205	1485.51	Malic acid, (3TMS)	Acid	1.25 ± 0.38	9.79 ± 3.42	4.49 ± 2.17	3.80 ± 0.91	11.40 ± 2.64
17	14.413	1760.63	Citric acid, (4TMS)	Acid	0.01 ± 0.00	0.07 ± 0.02	0.01 ± 0.01	0.00 ± 0.00	0.03 ± 0.00
**Total acids**					**10.25** ± **1.49**	**24.94** ± **8.33**	**15.75** ± **4.60**	**14.06** ± **4.07**	**29.12** ± **7.28**

18	5.003	1053.69	1,3-Propanediol, (2TMS)	Alcohol	0.39 ± 0.05	0.40 ± 0.05	0.34 ± 0.13	0.37 ± 0.06	0.39 ± 0.06
19	6.101	1121.97	Nonanol, (TMS)	Alcohol	0.07 ± 0.02	0.00 ± 0.00	0.01 ± 0.00	0.02 ± 0.01	0.02 ± 0.01
20	11.097	1476.75	Glycerol, (3TMS)	Alcohol	0.02 ± 0.01	0.18 ± 0.05	0.07 ± 0.07	0.11 ± 0.03	0.12 ± 0.03
**Total alcohols**					**0.48** ± **0.08**	**0.58** ± **0.10**	**0.41** ± **0.21**	**0.50** ± **0.10**	**0.53** ± **0.10**

21	5.483	1083.21	Valine, (TMS)	Amino acid	0.01 ± 0.01	0.00 ± 0.00	0.01 ± 0.00	0.01 ± 0.00	0.02 ± 0.00
22	5.76	1100.75	Alanine, (2TMS)	Amino acid	1.01 ± 0.32	0.12 ± 0.03	0.77 ± 0.12	0.33 ± 0.04	0.84 ± 0.25
23	6.514	1147.64	l-Leucine, (TMS)	Amino acid	0.08 ± 0.05	0.03 ± 0.01	0.04 ± 0.01	0.05 ± 0.01	0.10 ± 0.01
24	6.685	1158.24	l-Proline, (TMS)	Amino acid	0.01 ± 0.00	0.08 ± 0.02	0.02 ± 0.01	0.00 ± 0.00	0.05 ± 0.05
25	6.82	1166.97	Isoleucine, TMS	Amino acid	0.03 ± 0.02	0.01 ± 0.00	0.01 ± 0.00	0.01 ± 0.00	0.03 ± 0.01
26	7.057	1181.45	Methacryloyl glycine, (2TMS)	Amino acid	0.06 ± 0.01	0.10 ± 0.03	0.11 ± 0.03	0.06 ± 0.01	0.36 ± 0.15
27	7.349	1199.49	Tiglylglycine, (TMS)	Amino acid	0.15 ± 0.04	0.09 ± 0.07	0.09 ± 0.02	0.14 ± 0.03	0.19 ± 0.04
28	7.394	1202.53	Glycine 3TMS isomer	Amino acid	0.08 ± 0.01	0.07 ± 0.01	0.08 ± 0.00	0.08 ± 0.01	0.06 ± 0.01
29	7.464	1207.65	Valine, (2TMS)	Amino acid	1.62 ± 0.40	0.03 ± 0.00	0.62 ± 0.11	0.35 ± 0.08	0.70 ± 0.12
30	7.702	1224.79	γ-Hydroxybutyric acid, (2TMS)	Amino acid	0.42 ± 0.05	0.28 ± 0.01	0.25 ± 0.01	0.26 ± 0.07	0.25 ± 0.06
31	8.276	1265.93	l-Leucine, (TMS)	Amino acid	3.09 ± 0.73	0.06 ± 0.01	0.99 ± 0.17	0.75 ± 0.18	1.48 ± 0.26
32	8.59	1288.51	Isoleucine, (2TMS)	Amino acid	1.01 ± 0.23	0.03 ± 0.01	0.41 ± 0.08	0.24 ± 0.06	0.50 ± 0.09
33	8.62	1290.67	Proline, (di-TMS)	Amino acid	0.04 ± 0.01	3.75 ± 2.57	0.32 ± 0.10	0.03 ± 0.01	0.66 ± 0.89
34	8.768	1301.24	Glycine, (3TMS)	Amino acid	0.05 ± 0.02	0.01 ± 0.00	0.07 ± 0.03	0.02 ± 0.01	0.09 ± 0.02
35	9.54	1356.67	Serine, (3TMS)	Amino acid	0.39 ± 0.11	0.03 ± 0.01	0.37 ± 0.10	0.16 ± 0.04	0.31 ± 0.07
36	9.803	1375.56	*N*-Methylleucine, (2TMS)	Amino acid	0.48 ± 0.17	0.14 ± 0.01	0.06 ± 0.02	0.05 ± 0.01	0.19 ± 0.06
37	9.912	1383.36	l-Threonine, (3TMS)	Amino acid	0.39 ± 0.10	0.03 ± 0.01	0.26 ± 0.08	0.13 ± 0.04	0.36 ± 0.04
38	10.376	1418.66	β-Alanine, (3TMS)	Amino acid	0.03 ± 0.00	0.03 ± 0.00	0.03 ± 0.00	0.03 ± 0.01	0.03 ± 0.00
39	10.73	1447.39	Pyroglutamic acid, (2TMS)	Amino acid	0.09 ± 0.02	0.02 ± 0.00	0.02 ± 0.00	0.02 ± 0.00	0.04 ± 0.01
40	11.011	1470.14	Aspartic acid, (3TMS)	Amino acid	0.05 ± 0.03	0.01 ± 0.00	0.03 ± 0.02	0.02 ± 0.00	0.03 ± 0.00
41	11.574	1515.36	Pyroglutamic acid, (2TMS)	Amino acid	0.52 ± 0.13	0.10 ± 0.01	0.40 ± 0.05	0.14 ± 0.04	0.27 ± 0.46
42	11.594	1516.86	l-Aspartic acid, (3TMS)	Amino acid	0.09 ± 0.03	0.03 ± 0.01	0.51 ± 0.13	0.11 ± 0.02	0.60 ± 0.05
43	12.241	1568.96	α-Hydroxyglutaric acid, (3TMS)	Amino acid	0.01 ± 0.00	0.06 ± 0.01	0.01 ± 0.00	0.01 ± 0.00	0.02 ± 0.00
44	12.333	1577.06	Valine, (bis-TBDMS)	Amino acid	0.01 ± 0.00	0.01 ± 0.00	0.00 ± 0.00	0.01 ± 0.00	0.01 ± 0.00
45	12.693	1606.01	l-Aspartic acid, (3TMS)	Amino acid	0.03 ± 0.01	0.08 ± 0.01	0.06 ± 0.03	0.05 ± 0.01	0.13 ± 0.03
46	12.768	1612.69	Glutamic acid, (3TMS)	Amino acid	0.07 ± 0.04	0.03 ± 0.00	0.18 ± 0.12	0.04 ± 0.01	0.43 ± 0.14
47	12.863	1621.43	Phenylalanine, (2TMS)	Amino acid	0.05 ± 0.02	0.01 ± 0.00	0.01 ± 0.00	0.03 ± 0.01	0.01 ± 0.00
48	12.951	1629.15	Asparagine, (3TMS)	Amino acid	0.04 ± 0.01	0.02 ± 0.00	0.03 ± 0.00	0.01 ± 0.00	0.03 ± 0.01
49	13.139	1646.12	Valine, (bis-TBDMS)	Amino acid	0.14 ± 0.05	0.00 ± 0.00	0.00 ± 0.00	0.02 ± 0.01	0.01 ± 0.00
50	13.368	1666.55	l-Asparagine, (2TMS)	Amino acid	0.07 ± 0.02	0.01 ± 0.00	0.05 ± 0.01	0.01 ± 0.00	0.07 ± 0.01
51	14.232	1744.4	Isoleucine, (2TBDMS)	Amino acid	0.03 ± 0.01	0.01 ± 0.01	0.01 ± 0.00	0.01 ± 0.00	0.01 ± 0.00
52	16.212	1935.02	Tyrosine, (3TMS)	Amino acid	1.26 ± 0.40	0.02 ± 0.01	0.35 ± 0.11	0.23 ± 0.06	0.83 ± 0.14
**Total amino acids**					**11.41** ± **3.02**	**5.29** ± **2.88**	**6.17** ± **1.38**	**3.42** ± **0.78**	**8.70** ± **3.00**

53	5.649	1093.87	2-Ethylhexanoic acid, (TMS)	Fatty acid/ester	0.15 ± 0.02	0.14 ± 0.00	0.16 ± 0.02	0.16 ± 0.01	0.14 ± 0.02
54	11.406	1501.46	Methyl octanoate, (TMS)	Fatty acid/ester	0.25 ± 0.03	0.26 ± 0.03	0.27 ± 0.02	0.28 ± 0.04	0.26 ± 0.04
55	13.007	1634.29	Lauric acid, (TMS)	Fatty acid/ester	0.24 ± 0.11	0.10 ± 0.01	0.08 ± 0.01	0.14 ± 0.03	0.10 ± 0.01
56	17.072	2022.33	Palmitic acid, (TMS)	Fatty acid/ester	13.00 ± 3.19	5.24 ± 0.55	6.97 ± 1.18	9.05 ± 2.67	7.22 ± 0.78
57	18.596	2187.59	Linoleic acid, (TMS)	Fatty acid/ester	5.62 ± 2.06	1.65 ± 1.05	3.08 ± 0.96	3.06 ± 0.96	3.63 ± 0.18
58	18.641	2192.65	Oleic acid, (TMS)	Fatty acid/ester	4.54 ± 1.36	1.60 ± 1.07	1.68 ± 0.34	3.46 ± 1.04	1.94 ± 0.49
59	18.655	2194.97	α-Linolenic acid, (TMS)	Fatty acid/ester	10.48 ± 2.49	0.67 ± 0.43	1.14 ± 0.18	3.05 ± 0.90	2.19 ± 0.51
60	18.85	2216.82	Stearic acid, (TMS)	Fatty acid/ester	12.06 ± 2.65	4.96 ± 0.54	6.17 ± 0.59	7.39 ± 1.59	7.09 ± 0.19
61	20.391	2398.63	Octadecenoic acid	Fatty acid/ester	0.34 ± 0.07	0.10 ± 0.05	0.33 ± 0.22	0.14 ± 0.03	0.73 ± 0.30
62	21.34	2519.98	Heneicosanoic acid, (TBMS)	Fatty acid/ester	15.09 ± 1.97	11.95 ± 0.69	10.24 ± 4.81	13.61 ± 3.01	12.71 ± 2.87
63	21.717	2568.34	1-Monopalmitin, (TMS)	Fatty acid/ester	2.99 ± 1.12	1.13 ± 1.26	2.70 ± 1.72	3.21 ± 2.58	3.51 ± 4.15
64	22.155	2626.69	Octadecenoic acid, methyl ester, (3TMS)	Fatty acid/ester	1.24 ± 0.32	0.09 ± 0.02	0.14 ± 0.05	0.17 ± 0.05	0.11 ± 0.04
**Total fatty acid/ester**					**65.99** ± **15.38**	**27.90** ± **5.72**	**32.95** ± **10.10**	**43.71** ± **12.91**	**39.64** ± **9.56**

65	7.245	1193	2-Ketobutyric acid, enol, (2TMS)	Nitrogenous	0.04 ± 0.01	0.03 ± 0.00	0.03 ± 0.01	0.04 ± 0.01	0.03 ± 0.00
66	8.196	1260.18	Ethanolamine, (2TMS)	Nitrogenous	0.34 ± 0.11	0.04 ± 0.00	0.09 ± 0.03	0.13 ± 0.04	0.11 ± 0.03
67	8.498	1281.81	Nicotinic acid, (TMS)	Nitrogenous	0.09 ± 0.02	0.15 ± 0.02	0.12 ± 0.04	0.06 ± 0.00	0.22 ± 0.06
68	8.919	1312.2	Picolinic acid, (TMS)	Nitrogenous	0.01 ± 0.00	0.01 ± 0.00	0.01 ± 0.00	0.01 ± 0.00	0.01 ± 0.00
69	9.203	1332.45	Uracil, (2TMS)	Nitrogenous	0.00 ± 0.00	0.00 ± 0.00	0.00 ± 0.00	0.00 ± 0.00	0.03 ± 0.03
70	9.982	1388.34	Ethanolamine, (2TMS)	Nitrogenous	0.16 ± 0.02	0.14 ± 0.01	0.16 ± 0.01	0.17 ± 0.03	0.15 ± 0.02
71	11.348	1497	Trigonelline, (TMS)	Nitrogenous	0.02 ± 0.01	0.08 ± 0.03	0.03 ± 0.01	0.02 ± 0.00	0.11 ± 0.08
72	11.68	1523.69	γ-Aminobutyric acid, (3TMS)	Nitrogenous	0.86 ± 0.21	0.02 ± 0.00	0.54 ± 0.13	0.48 ± 0.14	0.57 ± 0.14
73	15.452	1859.65	Adenine, (2TMS)	Nitrogenous	0.27 ± 0.07	0.09 ± 0.01	0.07 ± 0.01	0.09 ± 0.02	0.12 ± 0.03
**Total nitrogenous**					**1.78** ± **0.44**	**0.55** ± **0.08**	**1.06** ± **0.24**	**0.99** ± **0.25**	**1.35** ± **0.38**

74	12.459	1586.48	d-Psicose, (5TMS)	Sugar	0.01 ± 0.00	0.01 ± 0.00	0.01 ± 0.00	0.02 ± 0.00	0.02 ± 0.00
75	12.843	1619.48	d-Arabinose, (4TMS)	Sugar	2.35 ± 0.78	0.63 ± 0.32	0.70 ± 0.21	0.87 ± 0.24	1.18 ± 0.17
76	12.985	1632.39	d-Mannose, (5TMS)	Sugar	0.19 ± 0.15	0.22 ± 0.16	0.18 ± 0.05	0.05 ± 0.01	0.14 ± 0.06
77	12.996	1633.26	l-Rhamnopyranose, (4TMS)	Sugar	0.03 ± 0.01	0.03 ± 0.02	0.03 ± 0.00	0.02 ± 0.00	0.05 ± 0.02
78	13.045	1637.56	d-Ribofuranose, (4TMS) (isomer 1)	Sugar	0.02 ± 0.01	0.04 ± 0.01	0.01 ± 0.00	0.03 ± 0.00	0.03 ± 0.01
79	13.202	1651.78	d-Arabinopyranose, (4TMS) (isomer 1)	Sugar	1.05 ± 0.52	1.18 ± 0.70	0.43 ± 0.20	0.87 ± 0.28	0.39 ± 0.08
80	13.414	1670.86	d-Arabinopyranose, (4TMS) (isomer 2)	Sugar	0.06 ± 0.02	0.06 ± 0.01	0.03 ± 0.01	0.05 ± 0.01	0.02 ± 0.01
81	13.511	1678.9	d-Ribofuranose, (4TMS) (isomer 2)	Sugar	0.21 ± 0.10	0.20 ± 0.13	0.09 ± 0.03	0.16 ± 0.06	0.10 ± 0.01
82	13.672	1693.95	Xylulose, (4TMS)	Sugar	0.13 ± 0.01	0.11 ± 0.01	0.12 ± 0.01	0.13 ± 0.02	0.11 ± 0.01
83	13.706	1696.07	d-Ribofuranose, (4TMS) (isomer 2)	Sugar	0.08 ± 0.02	0.02 ± 0.01	0.02 ± 0.01	0.02 ± 0.00	0.04 ± 0.01
84	13.745	1700.46	l-Rhamnopyranose, (4TMS)	Sugar	0.14 ± 0.09	0.14 ± 0.08	0.14 ± 0.05	0.06 ± 0.01	0.17 ± 0.08
85	13.8	1705.54	Arabose, (TMS)	Sugar	0.18 ± 0.07	0.01 ± 0.00	0.03 ± 0.03	0.04 ± 0.01	0.06 ± 0.01
86	13.915	1715.65	β-l-Fucopyranose, (4TMS)	Sugar	0.18 ± 0.08	0.21 ± 0.04	0.17 ± 0.10	0.28 ± 0.09	0.12 ± 0.04
87	13.928	1718.25	l-Rhamnopyranose, (4TMS)	Sugar	0.07 ± 0.02	0.01 ± 0.00	0.01 ± 0.00	0.02 ± 0.00	0.01 ± 0.00
88	14.072	1729.69	Ribitol, (5TMS)	Sugar	0.06 ± 0.02	0.01 ± 0.00	0.02 ± 0.01	0.02 ± 0.01	0.04 ± 0.01
89	14.313	1751.58	d-(+)-Galactose, (5TMS) (isomer 2)	Sugar	0.21 ± 0.08	0.34 ± 0.00	0.17 ± 0.06	1.13 ± 0.31	0.41 ± 0.10
90	14.483	1766.87	α-d-Mannopyranose, (5TMS)	Sugar	0.12 ± 0.08	0.23 ± 0.03	0.19 ± 0.11	0.28 ± 0.09	0.09 ± 0.03
91	14.522	1770.36	d-(−)-Ribofuranose, (4TMS) (isomer 2)	Sugar	0.29 ± 0.13	0.20 ± 0.01	0.36 ± 0.07	0.27 ± 0.05	0.29 ± 0.02
92	14.591	1776.49	L-Fucitol, (5TMS)	Sugar	0.11 ± 0.04	0.02 ± 0.00	0.03 ± 0.02	0.02 ± 0.00	0.05 ± 0.01
93	14.912	1806.01	l-Mannopyranose, 6-deoxy, (4TMS)	Sugar	2.73 ± 0.70	0.46 ± 0.18	0.71 ± 0.17	0.14 ± 0.04	1.88 ± 0.77
94	14.939	1808.5	d-tagatofuranose, (5TMS) (isomer 1)	Sugar	0.12 ± 0.04	0.25 ± 0.01	0.03 ± 0.01	0.03 ± 0.01	0.15 ± 0.06
95	14.987	1813.47	d-Fructofuranose, (5TMS) (isomer 1)	Sugar	0.72 ± 0.23	29.51 ± 2.90	3.83 ± 1.39	3.18 ± 1.31	3.57 ± 0.23
96	15.073	1821.98	d-Tagatofuranose, (5TMS) (isomer 2)	Sugar	2.18 ± 0.63	52.51 ± 3.72	9.55 ± 3.28	8.30 ± 2.69	9.39 ± 0.33
97	15.148	1829.44	d-Fructopyranose, (5TMS) (isomer 1)	Sugar	4.03 ± 1.03	66.22 ± 21.41	11.25 ± 3.00	8.98 ± 1.72	4.96 ± 0.97
98	15.388	1853.33	β-Galactofuranose, (5TMS)	Sugar	0.21 ± 0.09	4.14 ± 0.71	0.69 ± 0.40	0.39 ± 0.21	1.93 ± 0.81
99	15.408	1855.29	d-Talofuranose, (5TMS) (isomer 2)	Sugar	0.09 ± 0.04	0.03 ± 0.02	0.03 ± 0.01	0.03 ± 0.01	0.10 ± 0.03
100	15.477	1862.79	Sorbose, (5TMS)	Sugar	0.13 ± 0.04	2.73 ± 0.49	0.22 ± 0.07	0.18 ± 0.05	0.23 ± 0.01
101	15.554	1869.76	d-Galactopyranose, (5TMS) (isomer 1)	Sugar	1.99 ± 0.69	0.69 ± 0.17	0.79 ± 0.43	1.13 ± 0.29	0.27 ± 0.02
102	15.852	1899.29	d-Psicose, (5TMS)	Sugar	0.18 ± 0.13	5.78 ± 1.91	0.47 ± 0.17	0.37 ± 0.08	0.38 ± 0.12
103	15.896	1903.64	β-d-Mannopyranose, (5TMS)	Sugar	8.29 ± 2.34	93.50 ± 20.08	22.90 ± 8.01	13.59 ± 2.99	26.62 ± 1.69
104	16.008	1914.77	d-Glucose, 5TMS	Sugar	3.51 ± 1.21	0.59 ± 0.05	1.48 ± 0.75	1.94 ± 0.57	0.83 ± 0.08
105	16.03	1917.07	α-d-Talopyranose, (5TMS)	Sugar	0.44 ± 0.16	0.01 ± 0.00	0.09 ± 0.04	0.07 ± 0.03	0.26 ± 0.05
106	16.751	1988.52	β-d-Glucopyranose, (TMS)	Sugar	10.04 ± 2.78	102.49 ± 19.64	27.94 ± 8.80	15.66 ± 3.52	33.71 ± 2.69
107	17.275	2044.39	d-glucose, (5TMS)	Sugar	0.38 ± 0.19	0.13 ± 0.02	0.05 ± 0.02	0.06 ± 0.01	0.09 ± 0.02
108	18.74	2203.7	Maltose, (8TMS) (isomer 1)	Sugar	0.13 ± 0.04	1.15 ± 0.76	0.51 ± 0.38	0.08 ± 0.02	1.64 ± 1.20
109	20.09	2363.31	d-(+)-Cellobiose, (8TMS)	Sugar	0.05 ± 0.03	0.16 ± 0.07	0.10 ± 0.03	0.02 ± 0.01	0.22 ± 0.10
110	22.502	2674	Sucrose, (5TMS)	Sugar	0.69 ± 0.25	24.51 ± 15.70	54.96 ± 37.49	8.79 ± 2.21	113.87 ± 25.37
111	23.262	2779.72	d-Trehalose, (8TMS)	Sugar	0.03 ± 0.02	1.42 ± 0.58	0.07 ± 0.02	0.02 ± 0.01	0.09 ± 0.01
112	23.389	2796.3	3-α-Mannobiose, (8TMS) (isomer 2)	Sugar	0.26 ± 0.08	0.39 ± 0.06	0.26 ± 0.19	0.11 ± 0.02	0.19 ± 0.07
113	23.561	2813.35	Maltose, (8TMS) (isomer 1)	Sugar	1.31 ± 0.76	0.45 ± 0.05	0.17 ± 0.07	0.55 ± 0.15	0.47 ± 0.14
114	23.668	2823.39	d-Cellobiose, (8TMS) (isomer 1)	Sugar	1.12 ± 0.60	0.58 ± 0.03	0.22 ± 0.10	0.57 ± 0.15	0.78 ± 0.20
115	23.986	2852.92	3-α-Mannobiose, (8TMS) (isomer 1)	Sugar	0.16 ± 0.05	0.10 ± 0.01	0.06 ± 0.03	0.03 ± 0.00	0.17 ± 0.08
**Total sugar**					**44.25** ± **14.37**	**391.44** ± **90.10**	**139.13** ± **65.81**	**68.56** ± **17.28**	**205.13** ± **35.71**

116	9.746	1372.06	d-Erythronic acid γ-lactone, (2TMS)	Sugar acid	0.01 ± 0.00	0.01 ± 0.00	0.01 ± 0.01	0.01 ± 0.00	0.01 ± 0.00
117	11.974	1547.38	Erythronic acid, (4TMS)	Sugar acid	0.02 ± 0.01	0.04 ± 0.01	0.01 ± 0.00	0.01 ± 0.00	0.03 ± 0.00
118	12.182	1563.71	l-Threonic acid, (TMS)	Sugar acid	0.12 ± 0.02	0.56 ± 0.17	0.15 ± 0.06	0.56 ± 0.17	0.29 ± 0.05
119	12.563	1595.96	Xylonic acid, d-lactone, (TMS)	Sugar acid	0.02 ± 0.01	0.30 ± 0.13	0.02 ± 0.01	0.01 ± 0.01	0.02 ± 0.00
120	14.646	1781.63	Ribonic acid, (5TMS)	Sugar acid	0.04 ± 0.01	0.02 ± 0.00	0.02 ± 0.01	0.02 ± 0.01	0.02 ± 0.00
121	15.774	1891.56	Ribonic acid, (5TMS)	Sugar acid	0.59 ± 0.19	1.38 ± 0.39	0.62 ± 0.08	0.56 ± 0.11	0.98 ± 0.18
122	16.657	1979.89	Gulonic acid, 1,4-lactone, (4TMS)	Sugar acid	0.08 ± 0.02	0.86 ± 0.03	0.12 ± 0.05	0.13 ± 0.03	0.26 ± 0.04
123	17.003	2014.75	Glucaric acid, (6TMS)	Sugar acid	0.10 ± 0.01	0.08 ± 0.01	0.07 ± 0.03	0.08 ± 0.02	0.09 ± 0.01
124	17.162	2031.79	Glucaric acid, (6TMS)	Sugar acid	0.23 ± 0.13	1.04 ± 0.07	0.15 ± 0.05	0.64 ± 0.23	0.90 ± 0.07
125	20.254	2382.97	d-Glucuronic acid, (5TMS)	Sugar acid	0.13 ± 0.07	0.44 ± 0.07	0.15 ± 0.08	0.06 ± 0.02	0.34 ± 0.27
**Total sugar acid**					**1.35** ± **0.48**	**4.72** ± **0.88**	**1.33** ± **0.37**	**2.09** ± **0.60**	**2.95** ± **0.63**

126	8.339	1270.44	Glycerol, (tris-TMS)	Sugar alcohol	4.20 ± 0.86	5.18 ± 1.41	2.67 ± 0.39	2.75 ± 0.57	2.02 ± 0.12
127	11.52	1511.02	Erythritol, (4TMS)	Sugar alcohol	0.05 ± 0.03	0.08 ± 0.01	0.06 ± 0.02	0.01 ± 0.01	0.15 ± 0.09
128	11.938	1543.53	Pentitol, (4TMS)	Sugar alcohol	0.01 ± 0.00	0.01 ± 0.00	0.01 ± 0.00	0.01 ± 0.00	0.01 ± 0.00
129	15.305	1844.96	d-Pinitol, (5TMS)	Sugar alcohol	26.06 ± 4.12	18.19 ± 3.76	17.63 ± 2.70	19.20 ± 2.64	28.96 ± 3.59
130	16.275	1941.21	Sorbitol, (6TMS)	Sugar alcohol	0.04 ± 0.01	0.01 ± 0.00	0.02 ± 0.00	0.01 ± 0.00	0.02 ± 0.00
131	16.309	1944.64	d-Mannitol, (6TMS)	Sugar alcohol	0.12 ± 0.07	0.12 ± 0.02	0.06 ± 0.01	0.07 ± 0.02	0.10 ± 0.01
132	16.453	1959.06	d-Pinitol isomer, (5TMS)	Sugar alcohol	0.57 ± 0.23	0.53 ± 0.04	0.19 ± 0.06	0.18 ± 0.05	0.52 ± 0.11
133	16.544	1968.3	Myoinositol, (TMS)	Sugar alcohol	0.50 ± 0.15	2.44 ± 0.02	1.23 ± 0.56	0.48 ± 0.12	1.46 ± 0.27
134	16.964	2010.43	d-Pinitol isomer, (5TMS)	Sugar alcohol	0.40 ± 0.15	0.29 ± 0.01	0.20 ± 0.07	0.12 ± 0.03	0.44 ± 0.11
135	17.824	2104.18	Myoinositol, (TMS)	Sugar alcohol	0.88 ± 0.36	1.51 ± 0.40	2.48 ± 0.37	2.07 ± 0.51	1.93 ± 0.45
**Total sugar alcohol**					**32.82** ± **5.98**	**28.36** ± **5.68**	**24.54** ± **4.19**	**24.91** ± **3.95**	**35.62** ± **4.74**

#### 3.7.2. Supervised multivariate data analysis of *Balanites* GC-MS dataset

For better classification and discrimination of parts, supervised orthogonal partial least squares discriminant analysis (OPLS-DA) was adopted (Fig. S8A and S7B†) by constructing two respective models of the fruits (unripe *versus* the ripe) and the vegetative parts (leaves and stem) *versus* other parts. The fruit model showed higher prediction power with *Q*^2^ = 0.96 and *R*^2^ = 0.92 *versus* the vegetative parts model at 0.68 and 0.81, respectively. The respective loading *S*-plot (Fig. S8C†) revealed that monosaccharaides were richer in the ripe fruits *versus* disaccharides in unripe fruits, with a *p*-value < 0.05 and in agreement with PCA results. Likewise, loading the *S*-plot of the leaf and stem model against other parts (Fig. S7D†) revealed that sugars including mono- and disaccharides were higher in fruits parts than vegetative parts as expectedly, and in agreement with results presented in Table 4.

### 3.8. Comparison of ^1^H NMR, GC-MS and LC-MS multivariate PCA analysis of *Balanites* parts

By comparing the classification potential of the different profiling techniques in *Balanites viz*. LC-MS and ^1^H NMR from their respective PCA results revealed that the PCA score plot derived from LC-MS data (Fig. 4A) showed tighter clustering of data than that in whole region NMR spectra (Fig. 1A). With ^1^H NMR-based PCA, obvious segregation between *Balanites* leaf and stem from other parts could be observed alongside PC1. In contrast, PCA of LC-MS derived dataset showed a clear segregation pattern of the 5 parts along the 4 quadrants with seeds being most close to immature fruit metabolome clustering together and in agreement with NMR results. ^1^H NMR (Fig. 1E) revealed trigonelline being most enriched in immature fruit providing the highest level of the key alkaloid, whereas isorhamnetin was more associated with leaf leading to its segregation. While, the LC-MS loading plot (Fig. 4B) revealed that lipids *viz*. sphingolipid as well as a nitrogenous metabolite, pheophorbide A were more enriched in immature fruit, seed, and leaf parts. Steroidal saponins *viz*. diosgenin hdh and diosgenin hdhh major bioactive class in *Balanites* were found to be associated with *Balanites* mature fruit, and suggestive that fruit is the richest in diosgenin conjugates. Both NMR and LC-MS techniques were equally effective in classifying the *Balanites* parts and revealing different markers due to their different detection capacities. Owing to its better peak resolution and higher sensitivity, GC-MS can present another potential tool for profiling primary metabolites. The unsupervised PCA model (Fig. 7B) showed clear discrimination of ripe fruits from all other 4 parts and was in agreement with both NMR and LC-MS results.

### 3.9. Cytotoxic activity of *Balanites*' different parts

**Fig. 8:**
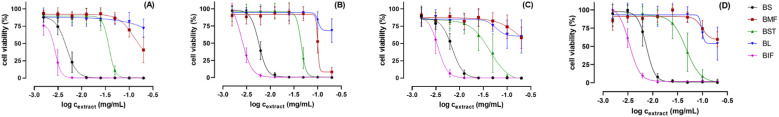
Dose–response curves of the viability of PC-3 cells (A and B) and HCT-116 cells (C and D) after 48 h treatment with the plant extracts using concentrations of 0–200 μg mL^−1^. The cells' viability was determined by using MTT assay (A and C) and CV assay (B and D), respectively. The data represent the mean of three independent biological replicates with at least technical duplicates ± SD.

Extracts of *Balanites*' different parts were further assessed for their *in vitro* cytotoxic effect against the human prostate cancer cell line (PC3) and the human colorectal cancer cell line (HCT-116) by using MTT and CV assays (see experimental), and in relation to metabolites profiling. Amongst the tested extracts, immature fruit (BIF) and seed (BS) exerted the most potent cytotoxic activity against both tested cell lines in both MTT and CV assays. BIF exhibited the strongest activity with IC_50_ values determined to be 2.8 and 3.4–3.5 μg mL^−1^, respectively, followed by (BS) with IC_50_ values of around 4.8–5.6 and 6.8–7.8 μg mL^−1^ against PC3 and HCT-116 cells, respectively (Table S1† and Fig. 8). These results are in line with the previously reported anticancer activity of trigonelline^56^ detected as a major metabolite in BIF, in addition to several known anticancer sphingolipids^57^ as major peaks in BIF and BS. *Balanites* stem extract (BST) showed moderate cytotoxicity with IC_50_ values of 38.3–45.6 and 47.7–48.2 μg mL^−1^ against PC3 and HCT-116 cells, respectively. In contrast, mature fruit extract (BMF) exhibited only weak activity against PC3 cells with corresponding higher IC_50_ values of 98.4–112.6 μg mL^−1^ and was inactive against HCT-116 cells. *Balanites* leaf extract (BL) was inactive against both cell lines at all tested doses (Table S1† and Fig. 8). Further detailed assays should be employed using isolated compounds to confirm the cytotoxic activity of the different parts *Balanites* and to identify the most active compound ingredients.

## 4. Conclusions

The current study presented the first comparative multiplex metabolomics-based investigation of *B. aegyptiaca* parts based on NMR, UPLC-MS, and GC-MS-based platforms coupled with multivariate analyses. NMR fingerprinting analysis led to the identification and quantification of 15 different metabolites including alkaloids, saponins, flavonoids, sugars, and amino and fatty acids. Furthermore, trigonelline and isorhamnetin accounted for BMF and BIF segregation in modeling results using NMR. In contrast, UPLC/MS/MS based on GNPS networking allowed for the annotation of 39 secondary metabolites mostly belonging to steroidal saponins (16), N-containing metabolites (4), phenolics (2), in addition to fatty acids and lipids (17). GC-MS profiling of primary metabolites identified a total of 135 peaks belonging to sugars, fatty acids/esters, amino acids, nitrogenous, and organic acids. Mature fruits were mostly predominated with monosaccharides, whereas disaccharides were more abundant in immature fruits. Leaf and stem were mostly enriched in amino acids and fatty acids. Detection of nicotinic acid, trigonelline as well as the sugar alcohol d-pinitol (first time to be detected) in *B. aegyptiaca* parts, especially in unripe fruit is suggestive of novel insight into the well-reported antidiabetic effect in *Balanites*. Moreover, *Balanites*' immature fruit and seeds exhibited potential efficacy against human prostate cancer (PC3) and human colorectal cancer (HCT-116) cell lines are likely attributed to its richness in trigonelline, diosgenin, and pheophorbide A, in addition to other bioactive metabolites. Nevertheless, it should be highlighted that these are only preliminary findings that will need to be followed by more extensive investigations of *B. aegyptiaca* organ extracts performing more advanced cancer cell-based *in vitro* and, ideally, *in vivo* studies, and identification and research on specific, isolated components.

## Author contributions

Mohamed A. Farag: conceptualization, methodology, writing – review, and editing. Mostafa H. Baky: investigation, data curation, formal analysis, writing – original draft. Ibrahim Morgan: biological investigation, writing – review and editing. Robert Rennert: biological investigation, writing – review and editing. Andrea Porzel: NMR analysis. Ludger A. Wessjohann: biological investigation, writing – review and editing. Magdy M. El-Sayed: investigation, writing – review and editing. Osama Gomaa: LCMS analysis and data acquisition. Mohamed Reda: GCMS analysis and data acquisition, Nehal S. Ramadan: investigation, data curation, formal analysis, writing – original draft.

## Conflicts of interest

There is no conflict of interest.

## Supplementary Material

RA-013-D3RA03141A-s001

## References

[cit1] Farag M. A., Porzel A., Wessjohann L. A. (2015). J. Pharm. Biomed. Anal..

[cit2] Baky M. H., Badawy M. T., Bakr A. F., Hegazi N. M., Abdellatif A., Farag M. A. (2021). RSC Adv..

[cit3] Murthy H. N., Yadav G. G., Dewir Y. H., Ibrahim A. (2020). Plants.

[cit4] Farag M. A., Porzel A., Wessjohann L. A. (2015). J. Pharm. Biomed. Anal..

[cit5] Chapagain B. P., Yehoshua Y., Wiesman Z. (2009). Bioresour. Technol..

[cit6] Sagna M. B., Diallo A., Sarr P. S., Ndiaye O., Goffner D., Guisse A. (2014). Afr. J. Biotechnol..

[cit7] Khamis G., Saleh A. M., Habeeb T. H., Hozzein W. N., Wadaan M. A., Papenbrock J., AbdElgawad H. (2020). J. Food Biochem..

[cit8] Farag M. A., Khattab A. R., Shamma S., Afifi S. M. J. F. (2021). Foods.

[cit9] Farag M. A., Khaled S. E., El Gingeehy Z., Shamma S. N., Zayed A. J. M. (2022). Metabolites.

[cit10] Farag M. A., Ramadan N. S., Shorbagi M., Farag N., Gad H. A. (2022). Foods.

[cit11] Otify A. M., Serag A., Porzel A., Wessjohann L. A., Farag M. A. (2022). Food Anal. Methods.

[cit12] Zayed A., Abdelwareth A., Mohamed T. A., Fahmy H. A., Porzel A., Wessjohann L. A., Farag M. A. J. F. C. (2022). Food Chem..

[cit13] Younis I. Y., Farag M. A., Elgamal A. M., Mohsen E. J. I. C. (2023). Ind. Crops Prod..

[cit14] Farag M. A., Shakour Z. T. A., Elmassry M. M., Donia M. S. J. F. C. (2022). Food Chem..

[cit15] Farag M. A., Hegazi N. M., Donia M. S. J. M. (2020). Metabolomics.

[cit16] Pluskal T., Castillo S., Villar-Briones A., Orešič M. (2010). BMC Bioinform..

[cit17] Reda E. H., Hegazi N. M., Marzouk M., Shakour Z. T. A., El-Halawany A. M., El-Kashoury E.-S. A., Mohamed T. A., Ibrahim M. A., Shams K. A., Abdel-Azim N. S. J. M. (2023). Molecules.

[cit18] Ernst M., Kang K. B., Caraballo-Rodríguez A. M., Nothias L.-F., Wandy J., Chen C., Wang M., Rogers S., Medema M. H., Dorrestein P. C. (2019). Metabolites.

[cit19] Farag M. A., Maamoun A. A., Ehrlich A., Fahmy S., Wesjohann L. A. (2017). LWT.

[cit20] Farag M. A., Ramadan N. S., Shorbagi M., Farag N., Gad H. A. J. F. (2022). Foods.

[cit21] Morgan I., Wessjohann L. A., Kaluđerović G. N. J. C. (2022). Cells.

[cit22] Lam Y. T., Ricardo M. G., Rennert R., Frolov A., Porzel A., Brandt W., Stark P., Westermann B., Arnold N. (2021). Int. J. Mol. Sci..

[cit23] Ware I., Franke K., Hussain H., Morgan I., Rennert R., Wessjohann L. A. J. M. (2022). Molecules.

[cit24] Feoktistova M., Geserick P., Leverkus M. (2016). Cold Spring Harbor Protoc..

[cit25] Otify A. M., El-Sayed A. M., Michel C. G., Farag M. A. (2019). Metabolomics.

[cit26] Wang X., Hong D.-F., Hu G.-L., Li Z.-R., Peng X.-R., Shi Q.-Q., Qiu M.-H. J. M. (2021). Molecules.

[cit27] Murthy H. N., Yadav G. G., Dewir Y. H., Ibrahim A. J. P. (2020). Plants.

[cit28] Choi M., Mukherjee S., Yun J. W. (2021). Phytother. Res..

[cit29] Hu Z., Wang C., Pan L., Han S., Jin M., Xiang Y., Zheng L., Li Z., Cao R., Qin B. (2021). Appl. Microbiol. Biotechnol..

[cit30] Pazhanichamy K., Bhuvaneswari K., Kunthavai B., Eevera T., Rajendran K. (2012). J. Planar Chromatogr.-Modern TLC.

[cit31] Kandil N. H., Ayoub I. M., El-Ahmady S. H., El-Moghazy S. A. (2022). Phytochem. Anal..

[cit32] Zaky A. S., Kandeil M., Abdel-Gabbar M., Fahmy E. M., Almehmadi M. M., Ali T. M., Ahmed O. M. J. P. (2022). Pharmaceutics.

[cit33] Gong G., Guan Y.-Y., Zhang Z.-L., Rahman K., Wang S.-J., Zhou S., Luan X., Zhang H. (2020). Biomed. Pharmacother..

[cit34] Osman S. M., El Kashak W. A., Wink M., El Raey M. A. (2016). Pharmacogn. Mag..

[cit35] Thelen J. J., Ohlrogge J. B. (2002). Metab. Eng..

[cit36] Du Q., Zhou L., Li M., Lyu F., Liu J., Ding Y. J. F. F. (2022). Food Front..

[cit37] Pontieri P., Troisi J., Calcagnile M., Bean S. R., Tilley M., Aramouni F., Boffa A., Pepe G., Campiglia P., Del Giudice F. J. F. (2022). Foods.

[cit38] Kim J., Jo Y., Cho D., Ryu D. J. N. C. (2022). Nat. Commun..

[cit39] Mousavi B., Azizi M.-H., Abbasi S. (2022). Food Chem. Mol. Sci..

[cit40] Serag A., Baky M. H., Döll S., Farag M. A. (2020). RSC Adv..

[cit41] Doğan H. O., Şenol O., Karadağ A., Yıldız S. N. (2022). Clin. Nutr. ESPEN.

[cit42] Cowan A. K. (2006). Plant Growth Regul..

[cit43] Sun M., Liu X., Gao H., Zhang B., Peng F., Xiao Y. (2022). Int. J. Mol. Sci..

[cit44] Völz R., Park J.-Y., Harris W., Hwang S., Lee Y.-H. (2021). BMC Biotechnol..

[cit45] Boubaker J., Bhouri W., Ben Sghaier M., Ghedira K., Dijoux Franca M., Chekir-Ghedira L. (2011). Cell Proliferation.

[cit46] Saide A., Lauritano C., Ianora A. J. M. D. (2020). Mar. Drugs.

[cit47] Baky M. H., Shamma S. N., Xiao J., Farag M. A. (2022). Food Chem..

[cit48] Bantle J. P. (2009). J. Nutr..

[cit49] Ramadan N. S., Wessjohann L. A., Mocan A., C Vodnar D., El-Sayed N. H., El-Toumy S. A., Abdou Mohamed D., Abdel Aziz Z., Ehrlich A., Farag M. J. M. A. (2020). Molecules.

[cit50] Sánchez-Hidalgo M., León-González A. J., Gálvez-Peralta M., González-Mauraza N. H., Martin-Cordero C. J. P. R. (2021). Phytochem. Rev..

[cit51] Tan H., Aziz A. A., Aroua M. (2013). Renew. Sustainable Energy Rev..

[cit52] Perez J. L., Jayaprakasha G., Yoo K. S., Patil B. S. (2008). J. Chromatogr. A.

[cit53] Farag M. A., Khaled S. E., El Gingeehy Z., Shamma S. N., Zayed A. (2022). Metabolites.

[cit54] Alagawany, Elnesr S. S., Farag M. R., Tiwari R., Yatoo M. I., Karthik K., Michalak I., Dhama K. J. V. Q. (2021). Vet. Quart..

[cit55] Yoshinari O., Igarashi K. (2010). Curr. Med. Chem..

[cit56] Mohamadi N., Sharififar F., Pournamdari M., Ansari M. (2018). J. Diet. Suppl..

[cit57] Padrón J. M. (2006). Curr. Med. Chem..

