# Supplementary material for: Comparison of Balanites aegyptiaca parts: metabolome providing insights into plant health benefits and valorization purposes as analyzed using multiplex GC-MS, LC-MS, NMR-based metabolomics, and molecular networking
Source: RSC Adv. 2023 Jul 20;13(31):21471–93. doi: 10.1039/d3ra03141a (PMC10359763; doi:10.1039/d3ra03141a)
Supplement: RA-013-D3RA03141A-s001 [file RA-013-D3RA03141A-s001.pdf]

Electronic Supplementary Material (ESI) for RSC Advances.
This journal is © The Royal Society of Chemistry 2023

Supplementary Material

Figure S1. ^{1}H NMR spectrum of *B. aegyptiaca* leaf extract in CD_3OD

Figure S2. A) OPLS-DA score plot. B) Loading S-plots derived from mature fruit modeled against immature fruit sample analyzed by 1H-NMR (δ 5.5-10.0 ppm), n = 3, showing the covariance p (1) against the correlation p(cor) (1) of the variables of the discriminating component of the OPLS-DA model. Cut-off values of p = 0.18 was used. C) Permutation plot. Designated variables are highlighted and identifications are discussed in the text

Figure S3. Base peak chromatograms (BPC) of five different organs of *Balanites aegypticae* in positive ionization mode.

Figure S4. Identification of diosgenin aglycone structure. a) fragmentation pathway scheme of diosgenin aglycone, b) MS/MS mirror match between the diosgenin in *Balanites aegyptica* and GNPS library spectrum, and c) Peak areas of diosgenin aglycone in different organs of *Balanites aegyptica*.

Figure S5. MS/MS fragmentation and abundance of compound **2 (**diosgenin-26- hexoside*) in different organs.

Figure S6. MS/MS fragmentation and abundance of compound **3** (diosgenin-3-hexoside).

Figure S7. GC–MS-based OPLS-DA score plot (a) derived from modeling silylated primary metabolites of Balanites aegyptiaca ripe fruit versus unripe fruit (n = 3). (b) Derived from modeling silylated primary metabolites of B.aegyptiaca leaf&stem versus other 3 organs(n = 3). (c) and (d)The respective loading S-plots showing the covariance p [1] against the correlation p(cor) [1] of the variables of the discriminating component of the OPLS-DA model. Cut-off values of $p < 0.519281$ was used. Designated variables are highlighted and identifications are discussed in the text

Table S1: IC_{50} values (μg/mL) of the plant extracts as determined for the PC-3 prostate cancer and HCT-116 colorectal cancer cell lines by performing MTT and CV assays, respectively.

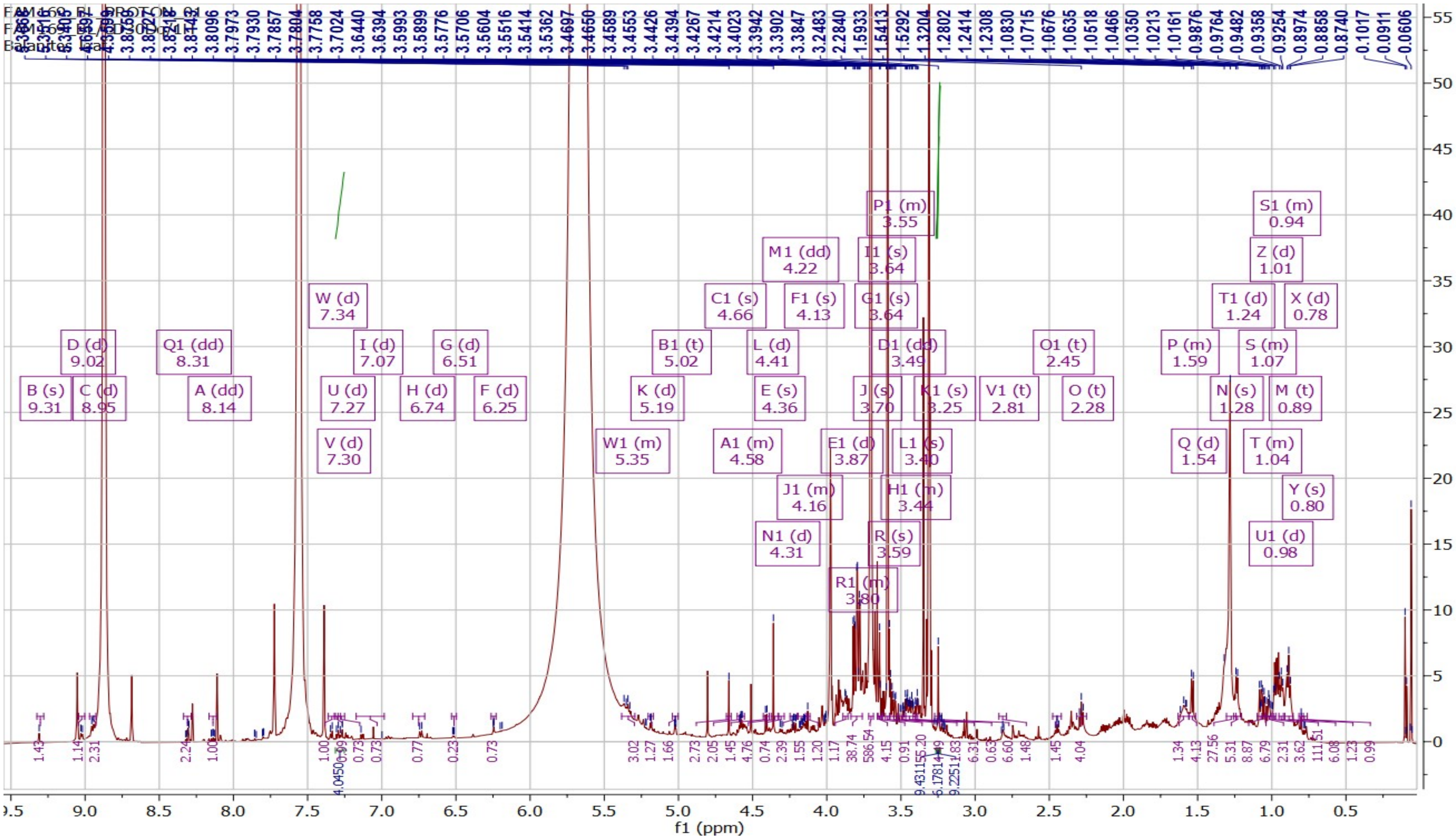

Fig. S1. ^{1}H NMR spectrum of *Balanites aegyptiaca* leaf extract

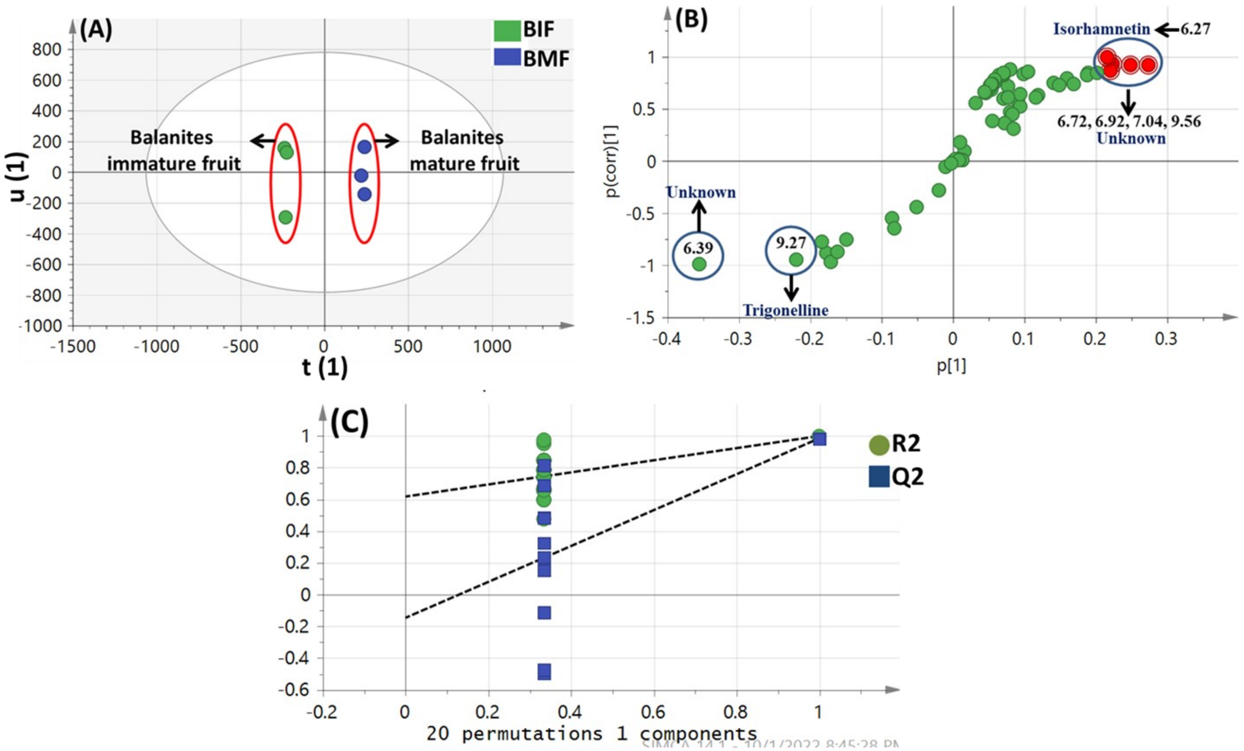

Figure S2. A) OPLS-DA score plot. B) Loading S-plots derived from mature fruit modeled against immature fruit sample analyzed by 1H-NMR (δ 5.5-10.0 ppm), n = 3, showing the covariance p (1) against the correlation p(cor) (1) of the variables of the discriminating component of the OPLS-DA model. Cut-off values of p = 0.18 was used. C) Permutation plot. Designated variables are highlighted and identifications are discussed in the text.

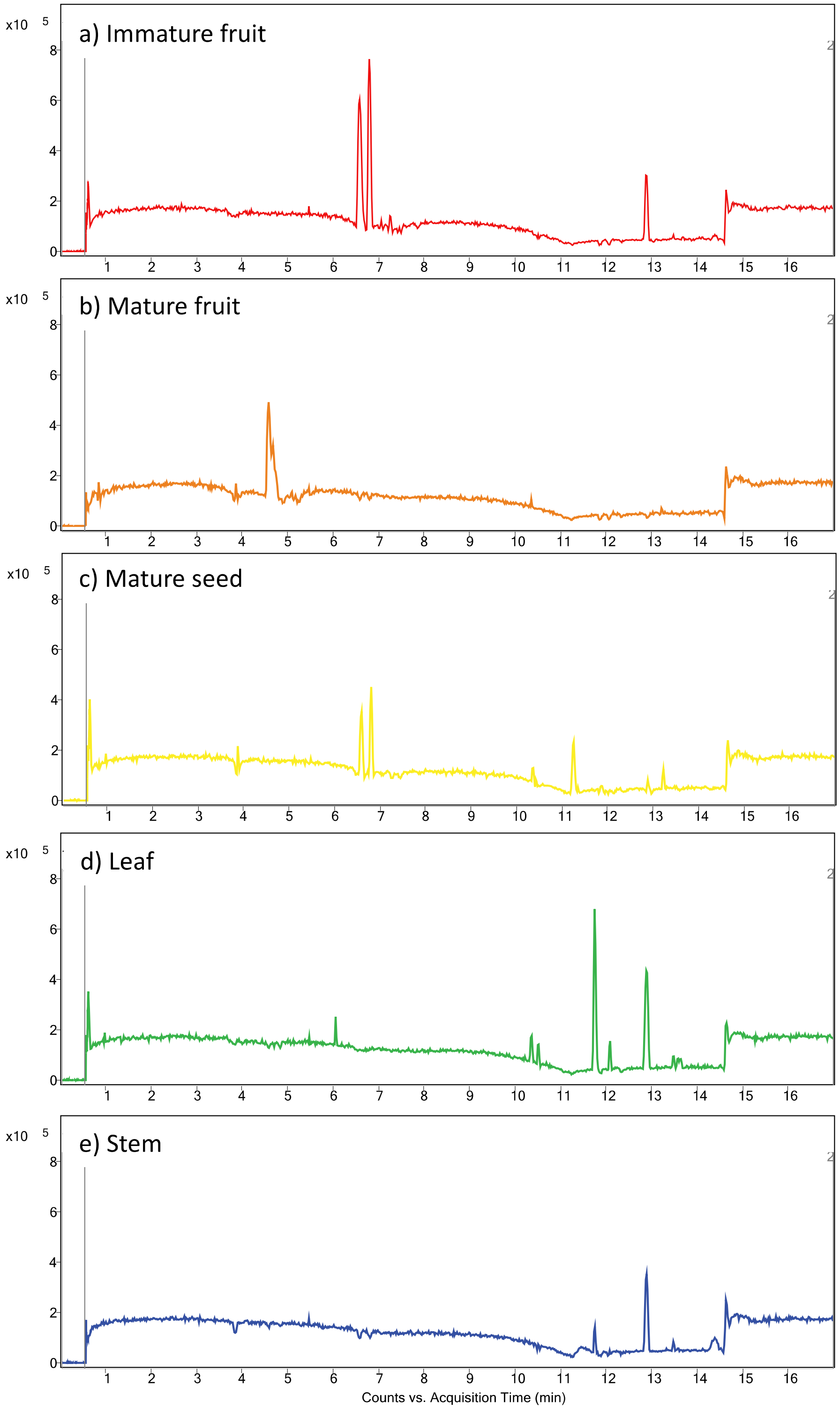

Figure S3. Base peak chromatograms (BPC) of five different organs of *Balanites aegypticae* in positive ionization mode.

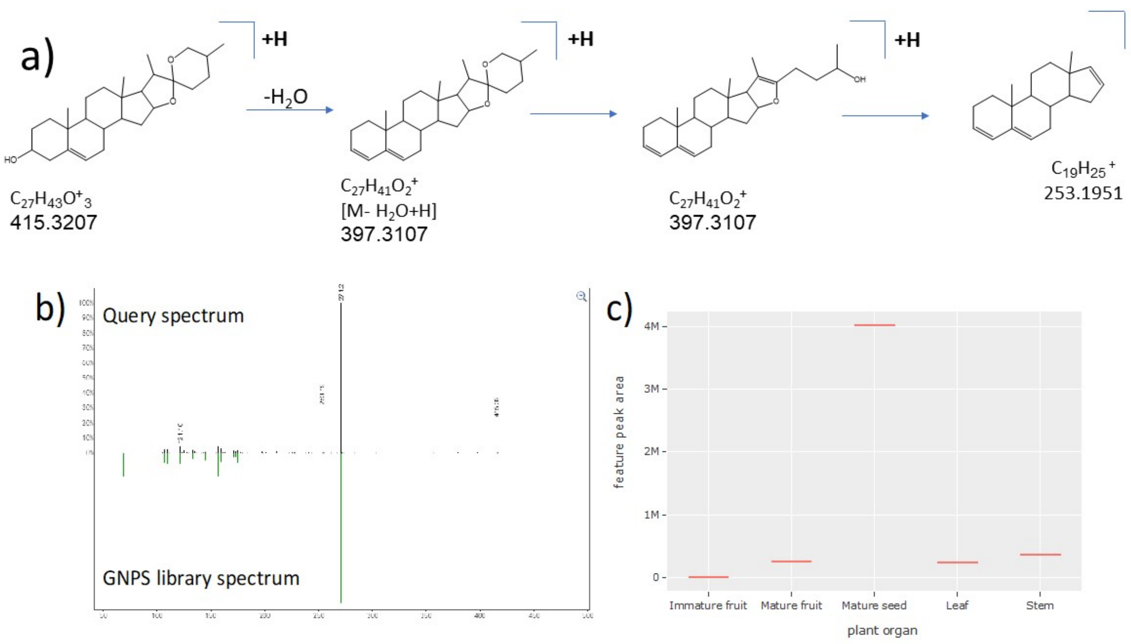

Figure S4. Identification of diosgenin aglycone structure. a) fragmentation pathway scheme of diosgenin aglycone, b) MS/MS mirror match between the diosgenin in *Balanites aegyptica* and GNPS library spectrum, and c) Peak areas of diosgenin aglycone in different organs of *Balanites aegyptica*.

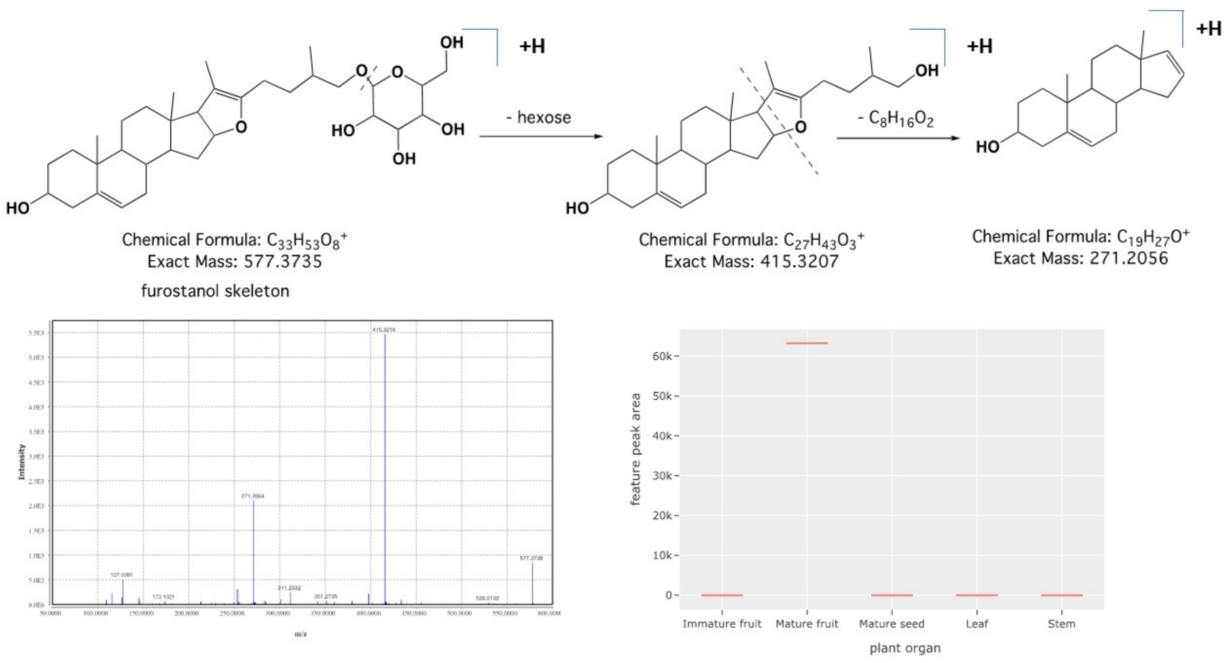

Figure S5. MS/MS fragmentation and abundance of compound **2** (diosgenin-26- hexoside*) in different organs.

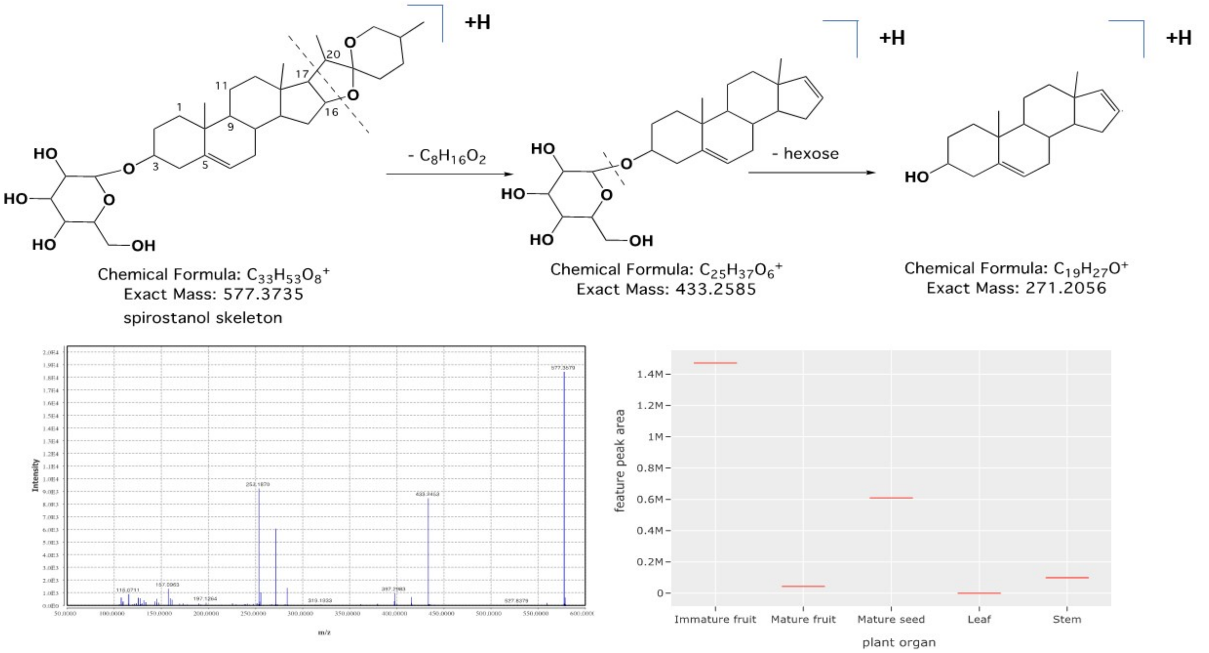

Figure S6. MS/MS fragmentation and abundance of compound **3** (diosgenin-3-hexoside).

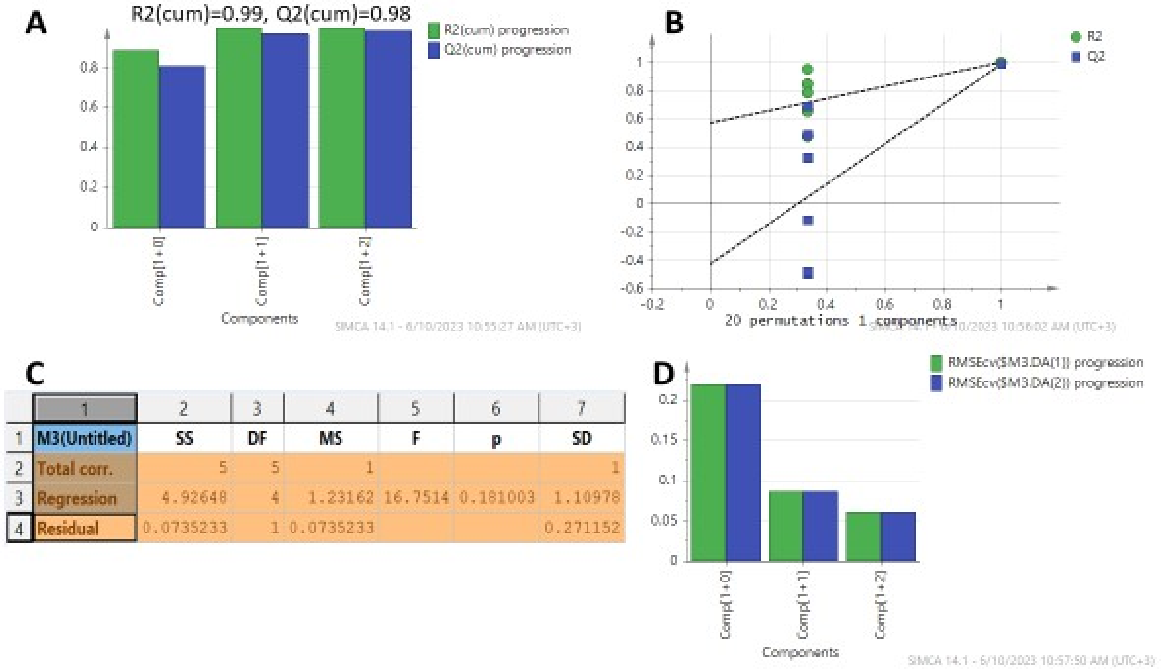

1	2	3	4	5	6	7
M3(Untitled)	SS	DF	MS	F	p	SD
Total corr.	5	5	1			1
Regression	4.92648	4	1.23162	16.7514	0.181003	1.10978
Residual	0.0735233	1	0.0735233			0.271152

Fig. S7. OPLS-DA model validation for modelling *B. aegyptiaca* samples based on LCMS in positive mode **A.** the diagnostic metrics R2 and Q2 **B.** permutation testing. n=20, and **C.** CV-ANOVA to assess for model statistical significance. **D.** SECV residuals.

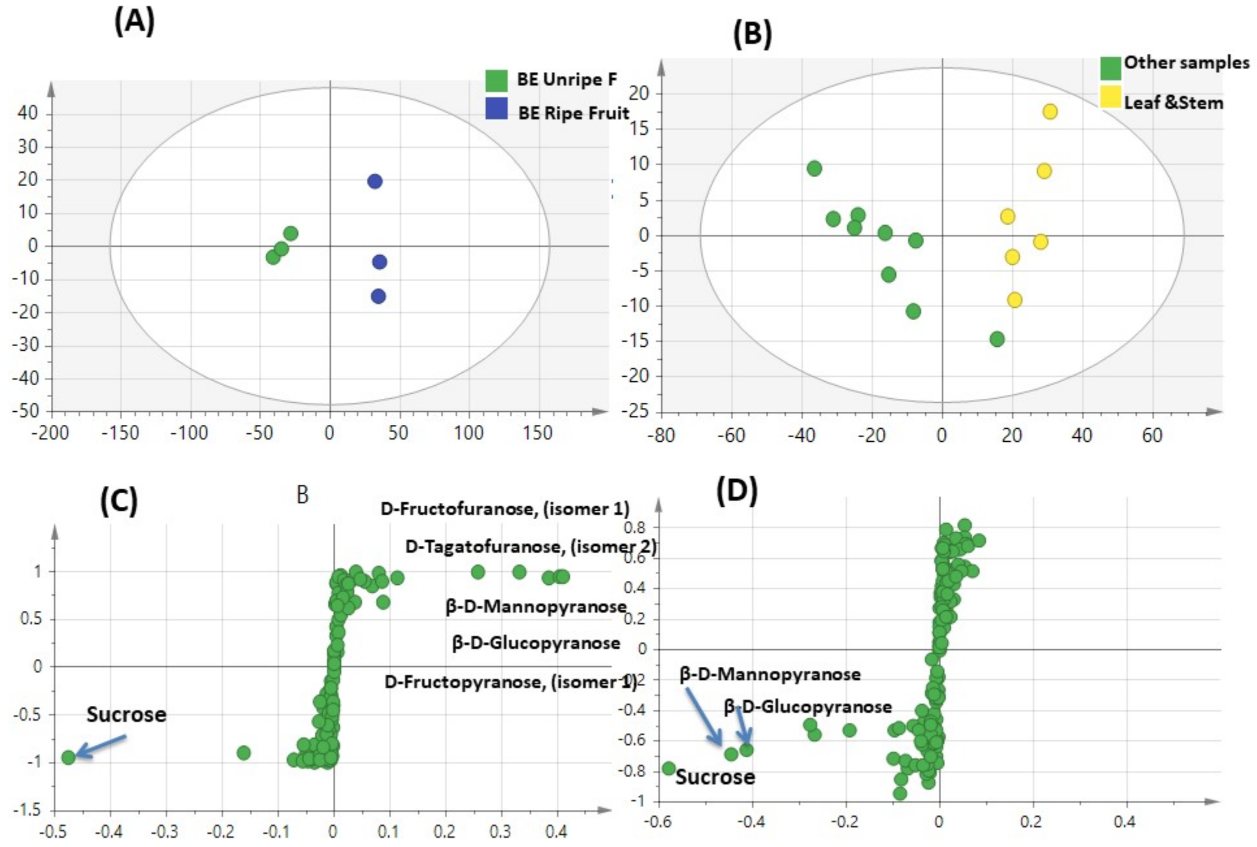

Figure S8. GC–MS-based OPLS-DA score plot (a) derived from modeling silylated primary metabolites of *Balanites aegyptiaca* ripe fruit versus unripe fruit (n = 3). (b) Derived from modeling silylated primary metabolites of *B.aegyptiaca* leaf&stem versus other 3 organs(n = 3). (c) and (d)The respective loading S-plots showing the covariance p [1] against the correlation p(cor) [1] of the variables of the discriminating component of the OPLS-DA model. Cut-off values of $p < 0.519281$ was used. Designated variables are highlighted and identifications are discussed in the text

Table S1: IC_{50} values (µg/mL) of the plant extracts as determined for the PC-3 prostate cancer and HCT-116 colorectal cancer cell lines by performing MTT and CV assays, respectively. The data represent the mean of three independent biological replicates with at least technical duplicates ± SD.

Relative IC_{50} (µg/mL)	**PC-3**		**HCT-116**	
	MTT assay	CV assay	MTT assay	CV assay
**BS**	4.8 ± 1.2	5.6 ± 0.6	6.8 ± 0.5	7.8 ± 1.2
**BMF**	98.4 ± 19.5	112.6 ± 19.1	> 200	> 200
**BST**	38.3 ± 7.3	45.6 ± 2.7	47.7 ± 22.0	48.2 ± 18.5
**BL**	> 200	> 200	> 200	> 200
**BIF**	2.8 ± 0.3	2.8 ± 0.3	3.4 ± 0.7	3.5 ± 0.4
